# Research on coordination of the NEV battery closed-loop supply chain considering CSR and fairness concerns in third-party recycling models

**DOI:** 10.1038/s41598-023-49047-8

**Published:** 2023-12-13

**Authors:** Zhenfang Zhang, Huan Liang

**Affiliations:** School of Economics and Management, Shanxi University, Taiyuan, 030006 China

**Keywords:** Environmental sciences, Environmental social sciences

## Abstract

Due to the pressure of the ecological environment and government, it is incumbent for enterprises to undertake corporate social responsibility (CSR). However, during the recycling process, awareness of equity concerns due to the distribution of benefits among members has intensified, and it is crucial to resolve channel conflicts and design a reasonable cooperation model to recycle used power batteries. Therefore, this paper constructs a closed-loop supply chain composed of power battery manufacturer, retailer and third-party collector based on the consideration of cascade utilization, in order to study the impact of the level of CSR and the degree of fairness concern on the decision-making of channel participants. Our research shows that: (1) Fairness concern behavior adversely affects the supply chain, which raises the sales price and reduces the collecting rate and the utility of the supply chain as a whole. (2) Undertaking CSR is beneficial to the development of the power battery market, and also helps to reduce the sense of unfairness among third-party recycling companies. (3) The cost-sharing contract effectively coordinates the distribution of supply chain benefits and improves the recycling rate. Finally, we further verify the correctness of the conclusions through numerical studies.

## Introduction

Under the policy support, new energy automobile industry is booming, but also through tax incentives, subsidies, index quotas, and other policies on new energy automobile enterprises to be tilted. Currently, China, the United States and Europe are the main drivers of global sales. Among them, China ranks first as a major producer and seller of new energy vehicles, accounting for about 60% of global electric vehicle sales. It is followed by Europe and the United States, with sales growing by 15% and 55% respectively in 2022. In addition, stimulated by incentive programs and supportive policies, electric vehicle sales in India, Thailand, and Indonesia have also achieved positive growth in recent years, with total sales of 80,000 volumes^1^. According to the IEA, global electric vehicle sales are forecast to reach 14 million units by the end of 2023, rose 35% year over year^2^.Since the new energy vehicles went on sale in 2009, the first batch of electric vehicles has traveled more than 200,000 km. Normally, due to performance and safety considerations, when the capacity of the electric vehicle battery drops to 70–80%, the electric vehicle has to replace the battery with a new one, and the current average life of the power battery is 5 to 10 years, which has also reached its service life^3,4^. According to the forecast of GGII, by 2025, the number of retired power batteries in China will reach 137.4 GWh, and the number of used batteries to be recycled will reach 960,000 tons^5,6^. Due to the large amount of metal elements such as lithium and cobalt and toxic electrolytes contained in retired NEV power batteries, they have high recycling value and hazardous properties^7^. The best technological route is to remanufacture retired batteries by testing and then carrying out gradient utilization or dismantling of raw materials^8^. Therefore, the recycling of used batteries is a huge business opportunity, but it is also accompanied by various techno-economic challenges such as imperfect collection policy, difficult recycling technology, and chaotic recycling system^9^. In addition, the existence of private "small workshops" has become the biggest competitor of the formal recycling enterprises, according to a report by the State Council of China, only 25% of the power batteries in China are recycled through formal channels^10^. The gross profit margin of these "small workshops" through their own special recycling channels is much higher than that of the professional recycling enterprises, but most of the "small workshops" only aim at making profits, do not have standardized recycling equipment, and do not have the technology to dismantle and recycle batteries, which makes it difficult for them to comply with the environmental protection requirements, so a large number of power batteries cannot be recycled. Therefore, a large number of power batteries cannot be recycled through formal channels. Coupled with the scrapping of new energy vehicles in the suburbs and recycling stations unattended, resulting in a serious waste of resources and environmental pollution should not be underestimated.

In response to the above problems, countries have introduced different policies to promote power battery recycling, for example, China issued the *Program for the Implementation of the Extended Producer Responsibility (EPR) System* in 2016, which requires producers to implement recycling, treatment and reuse of waste products and quantifies it as an important indicator for evaluating the level of corporate responsibility^11^, namely, Corporate Social Responsibility (CSR). On August 17, 2023, the new *EU Batteries Regulation* came into force, requiring expanded producer responsibility. Under the guidance of the government, more and more large corporations are actively developing social responsibility systems, such as Apple invests in wind energy-saving projects for its suppliers and bears part of the construction costs to improve the working environment. And companies that maximize self-interest are forced to transform or face bankruptcy. In 2019, for example, nine Chinese companies, including LIANMAO ELECTRONIC TECHNOLOGY Co., LTD., were asked to shut down production due to severe water pollution^12^. With the in-depth research on the concept of CSR, many scholars have also introduced CSR behaviors into the optimization and modeling of closed-loop supply chains, and confirmed that undertaking CSR is conducive to the enhancement of the value of supply chain enterprises^13–16^. For example, Zhang and Wang^17^ studied the relationship between competitive CSR and the government under different policies with incomplete information from the perspective of CSR and found that firms with higher levels of CSR receive more subsidies. On the contrary, higher tax rates for firms producing environmentally unfriendly products affect their own profits. Panda et al.^18^ defined CSR behavior as recycling used products for secondary use, stating that this behavior contributes to the improvement of the overall performance of the closed-loop supply chain.

In order to promote the secondary utilization of used power batteries, various electric vehicle battery collection and recycling programmes have been established in many countries. For example, Volkswagen require their EV customers to return used batteries to licensing points or local authority battery collection schemes^19^. In addition, some scholars enhance the recycling value of power batteries from the technical level^20^, and there are also studies from the perspective of the closed-loop supply chain, Liu et al.^21^ investigated the impact of uncertainty in the remaining capacity of decommissioned power batteries on the gradient utilization strategy, recycling decision and economic performance of the closed-loop supply chain. In 2018, Gu et al.^22^ optimized the overall profit of the supply chain by studying the pricing strategy based on the consideration of graded utilization. Later, in 2021, the impact of government subsidies on the stepwise utilization of retired power batteries was studied with other scholars^23^. Lou and other^24^ scholars compare the effectiveness of subsidizing by recycled volume and subsidizing by recycled battery capacity in improving the recycling rate of the closed-loop supply chain of new energy power batteries by establishing three recycling decision models. Zhong and Du^25^ also specified the government subsidy to each link from NEV from production to sales.

In addition, the choice of recycling model has been the subject of extensive research in supply chain management and operations, based on the three formal recycling routes that exist today^26^. Each country adopts different recycling models: ① Power battery manufacturers are responsible for recycling, such as South Korea's leading power battery company LG New Energy. ② Industry alliance recycling model (joint manufacturers) represented by Europe and the United States. ③ Third-party recycling enterprises are responsible for recycling, such as China's GEM Co., Ltd. Due to the special nature of power battery materials and the harmful effects on the environment, the decommissioned batteries after recycling need to be handled by more specialized enterprises. And some scholars also believe that, for example, Wang and Xia^27^ pointed out that electronic product recycling in the producer's extended responsibility system, the end product through the producer, the producer consortium or third-party recycling company from the consumer reverse return to the manufacturer, which the third-party logistics has an irreplaceable advantage. Xie et al.^28^ also pointed out that third-party recyclers possess specialized battery raw material recycling technology, which makes it a more reasonable choice for automobile manufacturers to transfer the recycled power batteries to third-party recyclers for processing.

Chronic disproportionality between inputs and revenues can create a sense of unfairness in a business. As early as 1986, a large number of behavioral economics scholars represented by Kahneman et al.^29^ found that people are very concerned about the fairness of the enterprise's earnings distribution. Existing studies on fairness concern mainly focus on its impact on system decision-making^30^, pricing decision-making^31,32^ and system coordination^33^. Zheng et al.^34^ compared the optimal solutions under five non-cooperative and cooperative models, and the results suggest that fairness concerns have an impact on upstream and downstream profit sharing. Wang et al.^35^ explored the impact of different attitudes of manufacturers under the fairness concern of third-party recyclers on the decision-making and profitability of various member firms in the supply chain, and found that a high level of fairness concern of third-party recyclers affects the magnitude of recycling rates. Yao and Teng^36^ introduced manufacturers' fairness concerns in the supply chain system of third-party recycling, and the results showed that regardless of whether the manufacturers have fairness concern behaviors or not, they can always obtain more profit channels, and at the same time, fairness concerns are always advantageous to their own utility maximization, and disadvantageous to other firms. Liu and two other scholars^37^ found that an increase in the degree of fairness concern will reduce the retail price of new power batteries and increase the recycling rate of retired batteries, and at the same time, the higher the price sensitivity of the cascade utilizer, the higher the utility of cascading efforts and the higher the cascading utilization rate. In addition, there are a large number of scholars coordinating on the adverse effects of fairness concerns on pricing decisions and supply chain decisions^38,39^. For example, Zou et al.^40^ explored the coordination of the benefit-sharing-cost-sharing contractual model on supply chains for closed-loop supply chains constituted by dual fairness concerns of the manufacturer and retailer. However, few scholars have addressed coordination under the third-party recycling model.

By combing through the above existing studies, it is not difficult to find that under the influence of government policies, the research on supply chain members' fulfillment of CSR and fairness concerns has already achieved certain results. Meanwhile, a large number of scholars in the existing research have also affirmed the necessity of recycling power batteries through third-party recycling channels by virtue of specialized recycling processing technology. The role of third-party recycling enterprises in the supply chain is becoming more and more prominent by virtue of specialized recycling processing technology. However, existing studies have considered the fair concern behavior of manufacturers or retailers, and although some articles have considered third-party collectors, they have only taken them as an influencing factor in the decision-making of other members. Few scholars have considered the fairness of profit sharing among third-party collectors arising from the high cost and low profitability of power battery testing and dismantling^41^. For example, China's leading third-party professional recycling company of power battery – GEM Co., Ltd, although the revenue rose rapidly with the whole environment, but the net profit is limited to improve. And the company's revenue showed negative growth in 2020 due to the impact of the epidemic. In addition, current articles involving third-party collectors only study the impact of behavioural decisions on profits and do not design a three-party coordination mechanism. Therefore, for the above problems, this paper mainly discusses the following issues:

1. Will power battery manufacturers taking on CSR have a positive impact on CLSC?
2. As one of the members of the closed-loop supply chain, how do the fair concern behaviors of third-party collector affect the decisions of other members?
3. How to design a cooperative mechanism to increase the collecting rate of decommissioned batteries, reduce environmental pollution and realize a "multiple win" in the closed-loop supply chain?

To address these issues, we construct a fair neutral game model and two extension models. One is that manufacturer have CSR. In the other, third-party collector engage in fairness concerns when manufacturer fulfill their CSR. The equilibrium decisions (wholesale price, retail price, purchase price, and collecting rate) were calculated for each model, as well as the profits of each participant. Then, the three models were compared to analyze the effects of CSR and fairness concern coefficients on CLSC, and finally the channel conflict problem was solved by contract.

## Problem assumptions and descriptions

### Problem assumptions

**Figure 1:**
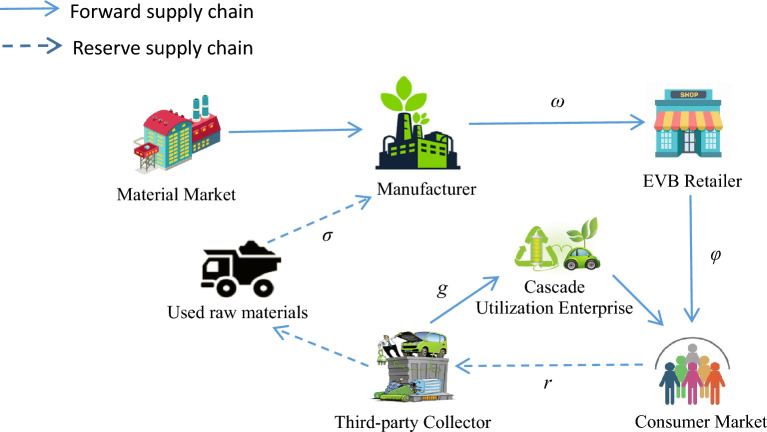
Power battery closed-loop supply chain model.

The closed-loop supply chain system in this paper consists of power battery manufacturer, retailer and third-party collector, (hereinafter referred to as M, R, and T, respectively), as shown in Fig. 1, and each member in the supply chain has different core businesses.

1. M is mainly responsible for the production of power batteries and sells the produced new energy power batteries to R. At the same time, the collecting business of power batteries is entrusted to T, which recovers the dismantled battery raw materials from the Tat a unit price *σ* and processes them into new batteries.
2. T is entrusted by the manufacturer to collect power batteries from consumers at a unit price *r*. After checking the battery capacity, it dismantles the raw materials of end-of-life batteries (with a capacity of less than 20%) and sells them to M, and sells the batteries with a capacity of 20–80% at a price of g to the cascade utilization enterprise for reuse.
3. R is an "intermediary" that does not have power battery production, recycling, dismantling and treatment technologies, and sells new energy power batteries at retail prices, publicizes them during the sales process, and provides corresponding services, such as customer service, warehousing, and after-sales service.

The relevant variables used in this paper are annotated as shown in Table 1.

**Table 1:** Description of relevant variables.

Parameter	Explanation
*c*_*m*_	Unit cost of production using new raw materials
*c*_*n*_	Unit cost of production using used raw materials
*∆*	Cost savings per unit produced from used raw materials (∆ = *C*_*m*_*–C*_*n*_)
*σ*	Unit cost of acquiring raw materials for power batteries from third party collector
***ω***	Power battery unit wholesale price
*φ*	Power battery unit retail price
*τ*	Collecting rate of used power battery
*λ*	Proportion of used power batteries dismantled for recycling of raw materials
*r*	Unit recycling price of used power battery
*g*	High energy density power battery unit wholesale price

In this closed-loop supply chain, there is a full-information Stackelberg game between supply chain members, in which M is the dominant player in the Stackelberg game, R and T are the followers, and the manufacturer and the R are fair-neutral firms, and only the T has fairness concern behavior. The third party is concerned about the M's profit and compares it with its own profit to analyze the "sense of fairness". The game leader formulates a strategy in advance based on the available information. The followers develop a strategy based on their own situation.

### Description of relevant assumptions

Based on the supply chain modeling and problem description, the following assumptions are made according to existing research:

#### Assumption 1

This paper takes M, R and T as the research subjects, and all are finite rationality and have a risk-averse attitude towards the power battery recycling problem^28^.

#### Assumption 2

In order to ensure the incentive of recycling, it is necessary to satisfy *σ* + *c*_*n*_ < *c*_*m*_, M has a certain cost advantage of using the raw materials acquired back from T to produce new power batteries. At the same time, in order to ensure the maximization of the manufacturer’s profit, M gives priority to the use of recycled materials for remanufacturing, and the cost of sorting, repairing and restructuring recycled materials is negligible.

#### Assumption 3

M's product made from recycled materials does not differ from the product made from new materials in terms of appearance, performance and so on, and they are sold in the same sales channel at the same price. Consumers have the same acceptance of the product made from different materials, so the retail price of R is the same.

#### Assumption 4

Referring to Panda^18^ and others, we assume that the market demand Q is a linear function of the retail price *φ*: $Q=\alpha -\beta \varphi$. In this model, *α* is the underlying market scale and *β* is the price sensitivity coefficient of demand.

#### Assumption 5

Collecting rate *τ* (0 < *τ* < 1) reflects the collector's effort to some extent, the higher the collecting effort, the higher the collecting rate. Referring to the assumptions of Hou et al.^37^ on collecting cost, there are $I=\frac{1}{2}A{\tau }^{2}$, where *I* is the fixed investment required by T to collect the product, A is the difficulty coefficient of collecting the EVBs, and A > 0.

## Basic modeling

Assuming that M prioritizes the use of recycled waste products as raw materials for the production of power batteries, and the average unit cost of production using recycled raw materials is *c*_*m*_ + *σ*, with a production success rate of 100%, and the average unit production cost of production using new materials for production is *c*_*m*_, the average unit manufacturing cost paid by M for the production of power batteries is:

$$C={c}_{m}\left(1-\lambda \tau \right)+\left({c}_{n}+\sigma \right)\lambda \tau ={c}_{m}-\lambda \tau \left(\Delta -\sigma \right).$$

Note that ∆ = *C*_*m*_*–C*_*n*_, and *∆* > *σ* > *r*, then the profit functions of the manufacturer, the EVB retailer, and the third-party collector are, respectively:

1 $${\pi }_{M}\left(\omega ,\sigma \right)=\left(\omega -{c}_{m}+\lambda \tau \left(\Delta -\sigma \right)\right)\left(\alpha -\beta \varphi \right)$$

2 $${\pi }_{R}\left(\varphi \right)=\left(\varphi -\omega \right)\left(\alpha -\beta \varphi \right)$$

3 $${\pi }_{T}\left(\tau \right)=\left(\sigma \lambda +g\left(1-\lambda \right)-r\right)\left(\alpha -\beta \varphi \right)\tau -\frac{1}{2}A{\tau }^{2}.$$

NEV production, as a developing industry, is bound to focus on social responsibility and make positive contributions to society, the environment and human beings; therefore, as a leader of the channel, M tends to have a certain awareness of social responsibility as well, and to consider in what way it should maximize the benefits of the society as the goal of its decision-making. In economic theory, the common interest of producers and consumers is the common interest of producers and consumers. Given the market demand, Consumer surplus (CS) refers to the difference between the maximum market price that consumers are willing to pay for a certain quantity of a product and the actual market price of the commodity^12^, therefore, consumer surplus can be expressed as:

$$CS={\int_{\varphi min}^{\varphi max}Dd\varphi} ={\int_{\frac{\alpha -D}{\beta }}^{\frac{\alpha }{\varphi }}\left(\alpha -\beta \varphi \right)d\varphi} =\frac{(\alpha -\beta \varphi {)}^{2}}{2\beta }.$$

Then the profit function when the manufacturer undertakes CSR is:

4 $${V}_{M}^{S}=\left(\omega -{c}_{m}+\lambda \tau \left(\Delta -\sigma \right)\right)\left(\alpha -\beta \varphi \right)+\frac{\theta (\alpha -\beta \varphi {)}^{2}}{2\beta }$$

where $\theta \in [\mathrm{0,1}]$, represents the degree of social responsibility undertaken by M. $\theta =0$ indicates that M is a pure profit maximizer and $\theta =1$ indicates that M is a full welfare maximizer. Since M has the social responsibility of recycling and remanufacturing, its profit function includes the pure profit obtained through the sale of EVB, the consumer surplus obtained through CSR practices, and the profit obtained from recycling and remanufacturing.

### Fairly neutral decentralized decision model (model TdMd)

When T is a fair-neutral enterprise, that is, T is a completely rational enterprise. In the closed-loop supply chain decision-making, T maximizes its own interests as the decision-making goal, and only pays attention to its own profit, without considering the difference between its own profit and the manufacturer's profit. At this time, the profit of the three parties is their utility, and the decision-making sequence of the game is as follows: ① M, as the dominant player, first determines the wholesale price *ω* and the purchase price *σ* of the power batteries. ② R and T, as the follower, make decisions at the same time: R determines the retail price *φ* according to the wholesale price *ω*, and T determines the collecting rate *τ* according to the repurchase price *r*.

#### Proposition 1

The profit function of M under decentralized decision-making is strictly concave with respect to *φ* and *ω* only if $A\ge \frac{\beta ((F+\lambda (\Delta -2\sigma ){)}^{2}-\lambda (\Delta -\sigma )(F-\lambda \sigma ))}{2}$, and the equilibrium outcomes according to the two-stage decision sequence are as follows (to make the expression simpler, let $F=r+g(\lambda -1)$, $J=\alpha -\beta {c}_{m}$):

$$\left\{\begin{array}{c}{\omega }_{d}^{*}=\frac{\alpha (-4A+\beta (F-\Delta \lambda {)}^{2})-4A\beta {c}_{m}}{\beta (-8A+\beta (F-\Delta \lambda {)}^{2})}\\ {\sigma }_{d}^{*}=\frac{F+\Delta \lambda }{2\lambda }\\ {\varphi }_{d}^{*}=\frac{\alpha }{2\beta }+\frac{\alpha (-4A+\beta (F-\Delta \lambda {)}^{2})-4A\beta {c}_{m}}{\beta (-16A+2\beta (F-\Delta \lambda {)}^{2})}.\\ {\tau }_{d}^{*}=\frac{(F-\Delta \lambda )J}{-8A+\beta (F-\Delta \lambda {)}^{2}}\\ {Q}_{d}^{*}=\frac{2AJ}{8A-\beta (F-\Delta \lambda {)}^{2}}\end{array}\right.$$

In addition, the profit function of each member of the closed-loop supply chain are:

$${\pi }_{dM}^{*}=-\frac{A{J}^{2}}{\beta (-8A+\beta (F-\Delta \lambda {)}^{2})}$$

$${\pi }_{dR}^{*}=\frac{4{A}^{2}{J}^{2}}{\beta (-8A+\beta (F-\Delta \lambda {)}^{2}{)}^{2}}$$

$${\pi }_{dT}^{*}=\frac{A(F-\Delta \lambda {)}^{2}{J}^{2}}{2(-8A+\beta (F-\Delta \lambda {)}^{2}{)}^{2}}$$

#### Proof

See Appendix A.

#### Theorem 1

In the model TdMd, there is ${\pi }_{dR}^{*}>{\pi }_{dM}^{*}>{\pi }_{dT}^{*}$.

#### Proof

See Appendix B.

From Theorem 1, we can get that in the decentralized supply chain, M occupies a dominant position, and in order to maximize the benefits, the manufacturer does not take into account the profits of downstream enterprises when determining the wholesale price of the power battery as well as the purchase price of used raw materials, which leads to the double marginalization of the utility, and substantially increases the wholesale price. Accordingly, in order to obtain higher profits, R sets the retail price based on M's wholesale price, increase the investment in marketing effort costs, and use more channels to increase market demand. Whereas the only way T can influence market demand to increase its profitability is to increased investment in efforts to collect EVBs, increase the collecting rate, and sell the recycled power batteries to cascade utilization enterprise or dismantled for raw materials, and thus the profit gained will be much less than that of the other firms.

#### Theorem 2

As the T' s collecting rate increases, its profit differential with the manufacturer increases, $\frac{\partial \Delta \pi }{\partial \tau }=A\tau >0$.

Since T sells raw metal materials dismantled from end-of-life batteries to M for remanufacturing, there is a direct link between their profits. we aim to respond to the impact on the collecting rate of used power batteries by comparing the profit difference between T and M. We set the profit difference between M and T to be $\Delta {\pi }_{d}={\pi }_{dM}^{*}-{\pi }_{dT}^{*}$. To determine the effect of *τ* on $\Delta {\pi }_{d}$, compute the first-order derivative of the difference in profits $\Delta {\pi }_{d}$ between M and T with respect to the recovery rate: $\frac{\partial \Delta \pi }{\partial \tau }=A\tau >0$. The result shows that $\Delta {\pi }_{d}$ increases as *τ* increases. This implies that T's collect effort is positively correlated with its profit difference with M. A rational T pursues its expected profit regardless of the profit difference. However, when a firm is irrational, for example, a strong sense of unfairness arises when a firm like GEM Co., Ltd spends a lot of effort to collect used power batteries and obtains not much profit. Then, in the third subsection, we will explore how T's equity concerns will have an impact in the supply chain decision-making process.

### Fairly neutral decentralized decision-making models under CSR (model TdMs)

In 1985, Vickers^45^ first demonstrated through a work that a firm's non-profit maximizing objective may be more beneficial than focusing only on profits^44^. In the context of a duopoly, he assumed that one firm had the single objective of maximizing profits while the other firm gave sales incentives, and the results showed that the incentivized firm made higher profits than its competitors. M uses the raw materials of dismantled batteries for remanufacturing to show its social responsibility, and this subsection analyzes the impact of CSR on the closed-loop supply chain of power batteries on the one hand, in order to validate Vickers’ theory, and on the other hand, the impact of CSR on the closed-loop supply chain of power batteries.

#### Proposition 2

In the case of a manufacturer with CSR, the manufacturer's profit function is a strictly concave function of *ω* and *σ*, when $A\ge -\frac{\beta (F-\Delta \lambda {)}^{2}}{2(\theta -4)}$, and the relevant results are as follows:

$$\left\{\begin{array}{c}{\omega }_{d}^{S*}=\frac{2A\alpha (\theta -2)+\alpha \beta (F-\Delta \lambda {)}^{2}-4A\beta {c}_{m}}{\beta (2A(\theta -4)+\beta (F-\Delta \lambda {)}^{2})}\\ {\sigma }_{d}^{S*}=\frac{F+\Delta \lambda }{2\lambda }\\ {\varphi }_{d}^{S*}=\frac{\alpha }{2\beta }+\frac{2A\alpha (\theta -2)+\alpha \beta (F-\Delta \lambda {)}^{2}-4A\beta {c}_{m}}{4A\beta (\theta -4)+2{\beta }^{2}(F-\Delta \lambda {)}^{2}}.\\ {\tau }_{d}^{S*}=\frac{(F-\Delta \lambda )J}{2A(\theta -4)+\beta (F-\Delta \lambda {)}^{2}}\\ {Q}_{d}^{S*}=-\frac{2AJ}{2A(\theta -4)+\beta (F-\Delta \lambda {)}^{2}}\end{array}\right.$$

Thus the profits of channel participants after M assumes CSR are:

$${V}_{M}^{S*}=-\frac{A{J}^{2}}{\beta (2A(\theta -4)+\beta (F-\Delta \lambda {)}^{2})}$$

$${V}_{R}^{S*}=\frac{4{A}^{2}{J}^{2}}{\beta (2A(\theta -4)+\beta (F-\Delta \lambda {)}^{2}{)}^{2}}$$

$${V}_{T}^{S*}=\frac{A(F-\Delta \lambda {)}^{2}{J}^{2}}{2(2A(\theta -4)+\beta (F-\Delta \lambda {)}^{2}{)}^{2}}$$

#### Proof

See Appendix C.

#### Theorem 3

In the model TdMs, we can obtain:

(a) $$\frac{\partial {V}_{M}^{S*}}{\partial \theta }>0$$, $$\frac{\partial {V}_{R}^{S*}}{\partial \theta }>0$$, $$\frac{\partial {V}_{T}^{S*}}{\partial \theta }>0$$

(b) $$\frac{\partial {\omega }_{d}^{S*}}{\partial \theta }<0$$, $$\frac{\partial {\sigma }_{d}^{S*}}{\partial \theta }=0$$, $$\frac{\partial {\varphi }_{d}^{S*}}{\partial \theta }<0$$, $$\frac{\partial {\tau }_{d}^{S*}}{\partial \theta }>0$$,$$\frac{\partial {Q}_{d}^{S*}}{\partial \theta }>0$$

#### Proof

See Appendix D.

When $\theta =0$, there are ${V}_{dM}^{S*}={\pi }_{dM}^{*}$, ${V}_{dR}^{S*}={\pi }_{dR}^{*}$, ${V}_{dT}^{S*}={\pi }_{dT}^{*}$, the profit of each subject is the profit when the manufacturer does not fulfill the CSR and T is fair neutral, after M undertakes the CSR, the profit of the three parties is all positively proportional to the CSR coefficient, which indicates that M taking the initiative to undertake CSR and promoting power battery collecting is conducive to improving the effectiveness of supply chain participants. Then the manufacturer is willing to undertake more CSR for its own profit, which coincidentally proves that the Vickers' theory. As the CSR coefficient increases, both the wholesale price and retail price of the unit show a decreasing trend, which is due to the fact that the CSR coefficient represents the social welfare that directly benefits the consumers, when the CSR level increases, consumers can buy the power battery at a lower price, which expands the market demand and facilitates T to collect more used power batteries. At the same time, we find that M's purchase price for used battery raw materials is not affected by CSR, and the reason for this phenomenon may be that the manufacturer improves T's profit by increasing the collecting rate of power batteries after assuming CSR, and because the profit of retailers and third-party collectors rises by a large margin, the manufacturer does not increase the purchase price to reduce the profit gap and save costs.

#### Theorem 4

The collecting rate of used batteries is proportional to the degree of CSR with a specific range under M assuming CSR:$$\left\{\begin{array}{c}{\overline{\tau }}_{d}^{S*}=\mathit{min}\left\{\frac{(F-\Delta \lambda )J}{-6A+\beta (F-\Delta \lambda {)}^{2}},1\right\}\\ {\underset{\_}{\tau }}_{d}^{S*}=\mathit{max}\left\{\frac{(F-\Delta \lambda )J}{-8A+\beta (F-\Delta \lambda {)}^{2}},0\right\}=\frac{(F-\Delta \lambda )J}{-8A+\beta (F-\Delta \lambda {)}^{2}}\end{array}\right.$$

From Theorem 3, we know that the collecting rate of used batteries increases with the CSR factor. It is not difficult to explain that in order to achieve profit growth, as a channel partner, when M performs more CSR, R needs to invest more sales effort to increase sales, and T helps the manufacturer to show higher social responsibility by collecting more batteries. Therefore, the recovery coefficient reaches a minimum when M is a pure profit maximizer, $\theta =0$, and a maximum when M becomes a full welfare maximizer, i.e. $\theta =1$, at which point the recovery coefficient may be greater than 1. This is obvious, because in the absence of CSR by the M, the M will not expend more effort to expand market demand and urge the T to make efforts to collect. However, the collect coefficient is bounded by 0 and 1. Therefore, there is a certain range of collecting rate in this model.

### Decentralized decision modeling for T fair concerns under CSR (model TfMs)

In the case of M's performance of CSR, the profits of all three parties are increased, but in the case of T, which has to invest a lot in the supply chain and is not subsidized, because the government generally subsidizes leading companies, and the only way to influence the market demand to enhance its profitability is to increase the collecting rate. From Theorem 1, the greater the rate of collecting of used batteries, the greater the gap in profits between T and M. Therefore, in order to maximize its own utility, T will pay more attention to its own profit in the whole supply chain. In this subsection, we will explore how T's fairness concern will affect the decision-making of each subject on the basis of M's CSR. Assume that denotes the fairness concern coefficient and ${\mu }_{0}>0$. The utility function of T is expressed as follows:

$${\mu }_{T}={\pi }_{T}-{\mu }_{0}\left({\pi }_{M}-{\pi }_{T}\right)=\left(1+{\mu }_{0}\right){\pi }_{T}-{\mu }_{0}{\pi }_{M}$$

Suppose ${U}_{T}=\frac{{\mu }_{T}}{1+{\mu }_{0}}={\pi }_{T}-\frac{{\mu }_{0}}{1+{\mu }_{0}}{\pi }_{M}$, and let $\mu =\frac{{\mu }_{0}}{1+{\mu }_{0}}$, the fair concern coefficient be re-expressed as *U*, and ${U}_{T}\in [\mathrm{0,1}]$, when ${\mu }_{0}=0$, $U=0$, T is fair neutral, and when ${\mu }_{0}\to \infty$, $U\to 1$, the larger its value represents the more unacceptable T is to distribute profits in the supply chain, and the stronger the fair concern is. Therefore, the utility function of T can be re-expressed as:

5 $$\begin{aligned} U_{T}^{S} & = \pi_{T} - \mu U_{M}^{S} \\ & = \left( {\sigma \lambda + g\left( {1 - \lambda } \right) - r} \right)\left( {\alpha - \beta \varphi } \right)\tau - \frac{1}{2}A\tau^{2} - \mu \left( {\left( {\omega - c_{m} + \lambda \tau \left( {\Delta - \sigma } \right)} \right)\left( {\alpha - \beta \varphi } \right) + \theta \frac{{(\alpha - \beta \varphi )^{2} }}{2\beta }} \right) \\ & = \left( {\alpha - \beta \varphi } \right)\left( {\left( {\sigma \lambda + g\left( {1 - \lambda } \right) - r} \right)\tau - \mu \left( {\lambda \tau \left( {\Delta - \sigma } \right) + \omega - c_{m} } \right)} \right) - \frac{1}{2}A\tau^{2} - \mu \theta \frac{{(\alpha - \beta \varphi )^{2} }}{2\beta }. \\ \end{aligned}$$

#### Proposition 3

The utility function of M under T fair concerns is a strictly concave function about only when $A\ge -\frac{{\beta }^{2}(F-\Delta \lambda {)}^{2}}{2(\theta -4)(1+\mu )}$. The equilibrium solution of the closed-loop supply chain is:

$$\left\{\begin{array}{c}{\omega }_{\mu }^{S*}=\frac{2A\alpha (\theta -2)(1+\mu )+\alpha \beta (F-\Delta \lambda {)}^{2}-4A\beta {c}_{m}(1+\mu )}{\beta (2A(\theta -4)(1+\mu )+\beta (F-\Delta \lambda {)}^{2})}\\ {\sigma }_{\mu }^{S*}=\frac{F+\Delta \lambda (1+2\mu )}{2\lambda (1+\mu )}\\ {\varphi }_{\mu }^{S*}=\frac{\alpha }{2\beta }+\frac{2A\alpha (\theta -2)(1+\mu )+\alpha \beta (F-\Delta \lambda {)}^{2}-4A\beta (1+\mu ){c}_{m}}{4A\beta (\theta -4)(1+\mu )+2{\beta }^{2}(F-\Delta \lambda {)}^{2}}.\\ {\tau }_{\mu }^{S*}=\frac{(F-\Delta \lambda )(1+\mu )J}{2A(\theta -4)(1+\mu )+\beta (F-\Delta \lambda {)}^{2}}\\ {Q}_{\mu }^{S*}=-\frac{2AJ(1+\mu )}{2A(\theta -4)(1+\mu )+\beta (F-\Delta \lambda {)}^{2}}\end{array}\right.$$

The utility of manufacturers, retailers, and third-party collector is:

$${U}_{M}^{S*}=-\frac{A\left(1+\mu \right){J}^{2}}{\beta (\beta (F-\Delta \lambda {)}^{2}+2A(\theta -4)(1+\mu ))}$$

$${U}_{R}^{S*}=\frac{4(1+\mu {)}^{2}{A}^{2}{J}^{2}}{\beta (2A(\theta -4)(1+\mu )+\beta (F-\Delta \lambda {)}^{2}{)}^{2}}$$

$${U}_{T}^{S*}=\frac{A(1+\mu ){J}^{2}((1+5\mu )\beta (F-\Delta \lambda {)}^{2}+4A(\theta -4)\mu (1+\mu ))}{2\beta (2A(\theta -4)(1+\mu )+\beta (F-\Delta \lambda {)}^{2}{)}^{2}}$$

#### Proof

See Appendix E.

#### Theorem 5

In the case where a manufacturer assumes corporate social responsibility and considers T fair concern, the following relationship is obtained by calculation:

(1) The effect of the degree of fairness concern coefficient on utility and optimal decision:

(a) $$\frac{\partial {U}_{M}^{S*}}{\partial \mu }<0$$, $$\frac{\partial {U}_{R}^{S*}}{\partial \mu }<0$$, $$\frac{\partial {U}_{T}^{S*}}{\partial \mu }<0$$, $$\frac{\partial \Delta U}{\partial \mu }>0$$

(b) $$\frac{\partial {\omega }_{\mu }^{S*}}{\partial \mu }>0$$, $$\frac{\partial {\sigma }_{\mu }^{S*}}{\partial \mu }>0$$, $$\frac{\partial {\varphi }_{\mu }^{S*}}{\partial \mu }>0$$, $$\frac{\partial {\tau }_{\mu }^{S*}}{\partial \mu }<0$$,$$\frac{\partial {Q}_{\mu }^{S*}}{\partial \mu }<0$$

(2) The effect of the degree of CSR on utility and optimal decision-making:

(a) $$\frac{\partial {U}_{M}^{S*}}{\partial \theta }>0$$, $$\frac{\partial {U}_{R}^{S*}}{\partial \theta }>0$$, $$\frac{\partial {U}_{T}^{S*}}{\partial \theta }>0$$

(b) $$\frac{\partial {\omega }_{\mu }^{S*}}{\partial \theta }<0$$, $$\frac{\partial {\sigma }_{\mu }^{S*}}{\partial \theta }=0$$, $$\frac{\partial {\varphi }_{\mu }^{S*}}{\partial \theta }<0$$, $$\frac{\partial {\tau }_{\mu }^{S*}}{\partial \theta }>0$$,$$\frac{\partial {Q}_{\mu }^{S*}}{\partial \theta }>0$$

#### Proof

See Appendix F.

When *μ* = 0, its equilibrium solution is the optimal solution when the model TdMs is fair and neutral, and in the presence of the fairness idea of T, the derivatives of the utility functions of M, R and T with respect to the coefficients of the fairness concern are less than 0. Obviously, the utility of the subjects in the supply chain and the utility of the whole supply chain decreases with the increase of the fairness concern of T, and at the same time, the difference of its utility with the manufacturer *∆U* is getting bigger and bigger. In addition, through Theorem 5 we can find that the wholesale price of power batteries, the retail price, and the repurchase price for used materials are positively related to the level of fairness concern, and T's collecting rate as well as market demand are negatively correlated with fairness concern, that is to say, as the coefficient of fairness concern enhances, and therefore raises the wholesale price to compensate for the cost depletion, and also leads to an increase in the price of power batteries to the extent that retailers reduce the cost of inputs for the level of service in order to protect their own interests. Ultimately, due to the "selfish" behavior of M, R, and T, the price of new energy vehicles is too high, and their promotion and service level will tend to be conservative, which directly reduces the willingness of consumers to buy, reduces demand, and allows other alternative products to have a greater chance of competition. M raises the purchase price for used materials in order to increase the incentive of third party collectors, and then this leads to a significant increase in the production cost. As a result, M will raise the wholesale price to compensate for the cost depletion, and also leads to an increase in the price of power batteries to the extent that retailers reduce the cost of inputs for the level of service in order to protect their own interests. Ultimately, due to the "selfish" behavior of M, R and T, the price of NEV is too high, and the promotion and level of service will tend to be conservative, which directly reduces the willingness to purchase, and reduces the demand. This directly reduces consumers' willingness to buy, reduces demand, and gives other alternative products a greater chance to compete. In short, this is a kind of behavior that is detrimental to others. After the M assumes CSR, the impact on the supply chain is similar to Theorem 3, and the final result is still favorable to the utility of the three parties, then, whether the gain brought by the CSR can make up for the loss brought by the T fair concern behavior still needs to be confirmed by numerical study.

## Coordination mechanism of the three partners under CSR (model TfMsc)

When T invests a large amount of recovery cost for recovery, its profit still accounts for a small percentage in the supply chain, so its sense of unfairness will be more and more intense, this paper adopts the cost-sharing mechanism to coordinate the closed-loop supply chain, so that the coordinated closed-loop supply chain system obtains more profits. The specific method is: M shares the recovery cost of part φ for T. According to this coordination mechanism, the profit function of each member of the system can be obtained as follows:

6 $${U}_{\phi M}^{S}=\left(\omega -{c}_{m}+\lambda \tau \left(\Delta -\sigma \right)\right)\left(\alpha -\beta \varphi \right)+\frac{\theta (\alpha -\beta \varphi {)}^{2}}{2\beta }-\phi \frac{1}{2}A{\tau }^{2}$$

7 $${U}_{\phi R}^{S}=\left(\varphi -\omega \right)\left(\alpha -\beta \varphi \right)$$

8 $${U}_{\phi M}^{S}=(\left(\sigma \lambda +g\left(1-\lambda \right)-r\right)\tau -\mu \left(\omega -{c}_{m}+\lambda \tau \left(\Delta -\sigma \right)\right)D-\mu \theta \frac{{D}^{2}}{2\beta }-\frac{1}{2}\left(1-\phi -\mu \phi \right)A{\tau }^{2}$$

### Proposition 4

The equilibrium solution obtained after M bears part of the recovery cost for T is as follows:

$$\left\{\begin{array}{c}{\omega }_{\phi \mu }^{S*}=\frac{\alpha \beta (F-\Delta \lambda {)}^{2}-(A\alpha (\theta -2)-2A\beta {c}_{m})(1+\mu )(\phi -\mu \phi -2)}{\beta (-A(\theta -4)(1+\mu )(\phi -\mu \phi -2)+\beta (F-\Delta \lambda {)}^{2})}\\ {\sigma }_{\mu }^{S*}=\Delta -\frac{F-\Delta \lambda }{\lambda \left(1+\mu \right)\left(\phi -\mu \phi -2\right)}\\ {\varphi }_{\mu }^{S*}=\frac{\alpha }{2\beta }+\frac{2A\alpha (\theta -2)(1+\mu )+\alpha \beta (F-\Delta \lambda {)}^{2}-4A\beta (1+\mu ){c}_{m}}{2\beta (-A(\theta -4)(1+\mu )(\phi -\mu \phi -2)+\beta (F-\Delta \lambda {)}^{2})}\\ {\tau }_{\mu }^{S*}=\frac{\left(F-\Delta \lambda \right)\left(1+\mu \right)J}{-A(\theta -4)(1+\mu )(\phi -\mu \phi -2)+\beta (F-\Delta \lambda {)}^{2}}\\ {Q}_{\mu }^{S*}=\frac{2AJ\left(1+\mu \right)\left(\phi -\mu \phi -2\right)}{-A(\theta -4)(1+\mu )(\phi -\mu \phi -2)+\beta (F-\Delta \lambda {)}^{2}}\end{array}\right.$$

Therefore, the utility of the three parties is:

$${U}_{\phi M}^{S*}=\frac{A\left(1+\mu \right)\left(\phi -\mu \phi -2\right){J}^{2}}{2\beta (\beta (F-\Delta \lambda {)}^{2}-A(\theta -4)(1+\mu )(\phi -\mu \phi -2))}$$

$${U}_{\phi R}^{S*}=\frac{((1+\mu )AJ(\phi -\mu \phi -2){)}^{2}}{\beta (-A(\theta -4)(1+\mu )(\phi -\mu \phi -2)+\beta (F-\Delta \lambda {)}^{2}{)}^{2}}$$

$${U}_{\phi T}^{S*}=\frac{A(1+\mu ){J}^{2}(A\theta \mu (1+\mu )(\phi -\mu \phi -2{)}^{2}+\beta (F-\Delta \lambda {)}^{2}{C}_{1}-2\mu ({C}_{2}-\beta {C}_{3}))}{2\beta (-A(\theta -4)(1+\mu )(\phi -\mu \phi -2)+\beta (F-\Delta \lambda {)}^{2}{)}^{2}}$$

### Proof

See Appendix G.

In order to realize the effectiveness of coordination, the profit of the three parties after the coordination of the cost-sharing contract has to be greater than the profit under decentralized decision-making in which T considers equity, it has to satisfy the ${U}_{\phi M}^{S*}>{U}_{M}^{S*}$, ${U}_{\phi R}^{S*}>{U}_{R}^{S*}$, ${U}_{\phi T}^{S*}>{U}_{T}^{S*}$, By taking the smallest of these values, the result is ${U}_{\phi M}^{S*}>{U}_{M}^{S*}$. After solving, the range of values of the cost-sharing ratio is: $0<{\phi }_{1}<\frac{\beta (F-\Delta \lambda {)}^{2}+2A(\theta -4)(1+\mu )}{A(\theta -4)(1+\mu {)}^{2}}$. In Theorem 3, we learn that it is possible for M to have a recovery rate greater than 1 when it fulfills its social responsibility, so that when $0<\tau <1$, there is, $0<{\phi }_{2}<\frac{2A(\theta -4)(1+\mu )-(1+\mu )(F-\Delta \lambda )J+\beta (F-\Delta \lambda {)}^{2}}{A(\theta -4)(1+\mu {)}^{2}}$, and because of ${\phi }_{1}-{\phi }_{2}>0$, the final solution for the cost-sharing ratio is $0<{\phi }_{2}<\frac{2A(\theta -4)(1+\mu )-(1+\mu )(F-\Delta \lambda )J+\beta (F-\Delta \lambda {)}^{2}}{A(\theta -4)(1+\mu {)}^{2}}$. Taking the partial derivative of the upper limit of the cost-sharing coefficient with respect to *µ*, we obtain $\frac{\partial \overline{{\phi }_{2}}}{\partial \mu }=-\frac{2A(\theta -4)(1+\mu )-(1+\mu )(F-\Delta \lambda )J+2\beta (F-\Delta \lambda {)}^{2}}{A(\theta -4)(1+\mu {)}^{3}}>0$, It can be seen that when *μ* gradually increases, the upper bound of the coordination parameter also gradually increases, and the results of the study show that the more importance manufacturers attach to fairness, the stronger their bargaining power and the larger the cost-sharing proportion they obtain. And M, as a cost-sharing party, should consider the most favorable sharing ratio of its own profit under the premise of compensating T profit. In this case, it can effectively reduce the damage of fairness concerns to the green CLSC, maintain and promote cooperation among each other, promote consumption, reduce resource waste, protect the environment, and form a green economic model that is friendly and harmonious between the economy and the environment.

## Numerical study

This subsection obtains the trajectory graphs of each decision variable of the three parties as well as the profit by assigning parameters and using Matlab software. Analyzing the equilibrium results of the game participants of the parties based on the T-fair concern visually verifies the conclusions drawn in the previous section. Similar to the related research^35^ and combined with the actual situation, the parameters are set as in Table 2.

**Table 2:** Table of simulation values.

Parameters	*c*_*m*_	*A*	*α*	*g*	*λ*	*r*	*β*	*θ*
Value	70	1200	1200	60	0.4	36	15	0.8

Numerical study investigate the impact of the fairness concern factor and the degree of CSR on wholesale price, repurchase price, retail price, collecting rate, and market demand. The red surface, yellow surface and green surface in the figure below represent the equilibrium results of the fairness-neutral decentralized decision-making model (TdMd) as well as the fairness-neutral TdMs model and the fairness-concerned TfMs model with CSR, respectively.

### Impact of fairness and CSR coefficient on M decision making

**Figure 2:**
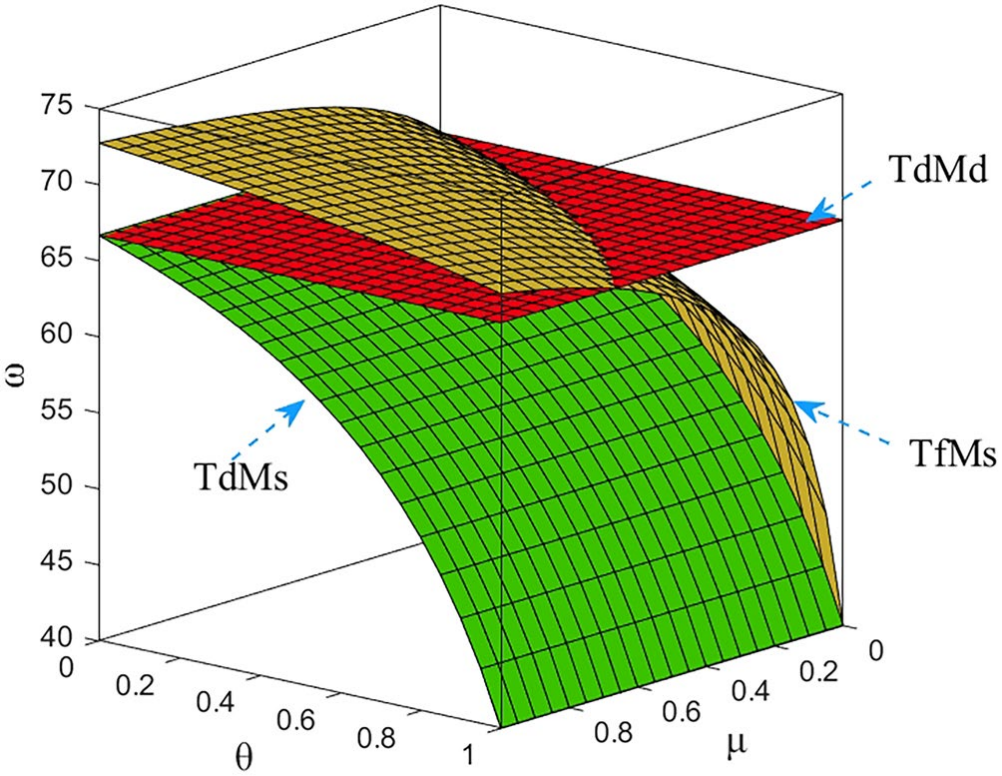
The impact of *μ* and *θ* on wholesale price.

**Figure 3:**
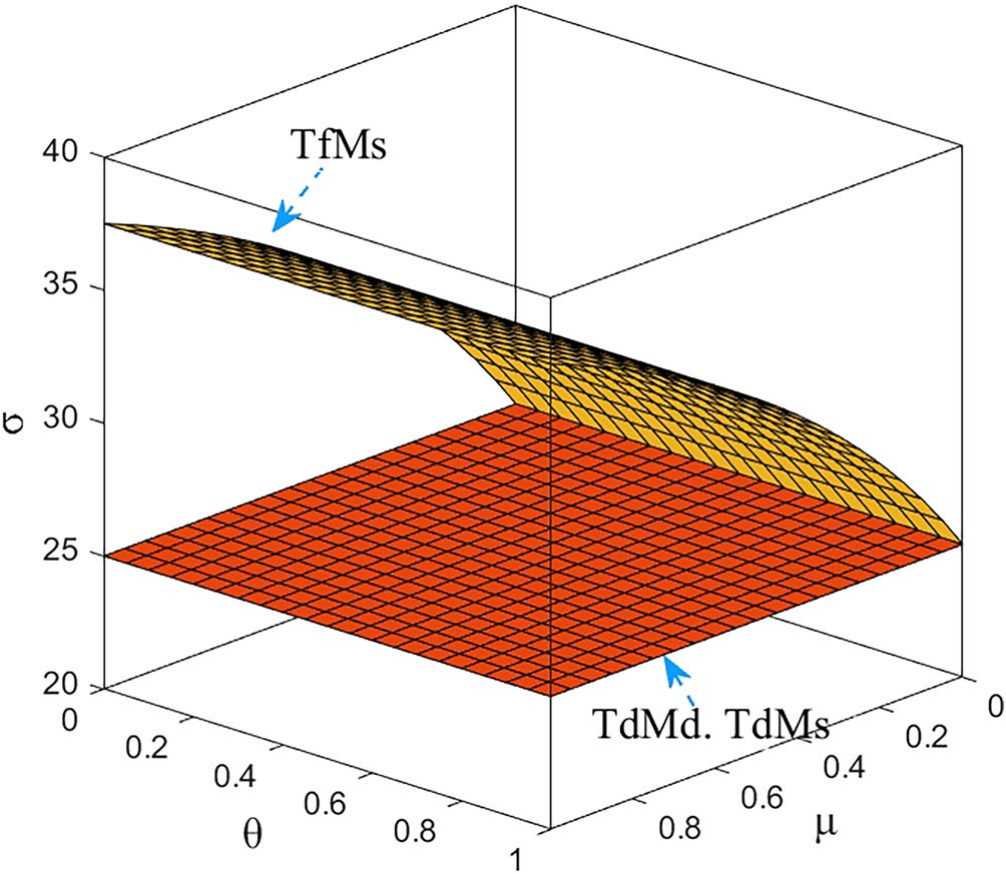
The impact of *μ* and *θ* on repurchase price.

In Figs. 2, 3, it is clear that about the M equalization results for the three cases show different variations. It can be seen from the simulation results. The equilibrium results of the other two models are changed on the basis of the absence of CSR as well as the sense of fairness, and when the manufacturer assumes CSR, it reduces the wholesale price, the selling price of EVBs, and the impact on the wholesale price is more obvious than the impact on the selling price, and the decrease is greater. At the same time, through Fig. 3 we find that the purchase price of the raw materials of used power batteries it collects from the third-party collector does not change, which indicates that the change of the purchase price has no effect on whether M fulfills its social responsibility, which further verifies the result of Theorem 3. When T starts to be dissatisfied with the profit distribution, the collection rate of used power batteries gradually decreases, the metal raw materials dismantled to become less, so M needs to buy expensive new raw materials from the raw material market, the manufacturing cost increases, and the price of power batteries gradually rises. In order to reduce the sense of unfairness for third-party collectors, M then raises the price of the raw metal materials of the used batteries it acquires from T. Finally, it is also confirmed numerically that when the T fair concern coefficient reaches a certain value, CSR can not only compensate for part of the loss caused by T fair concern to the supply chain and optimize the supply chain system, but also perform more competitively than pure profit maximization. Here, the manufacturer's intention is not to coordinate the channel, but to encourage retailers to sell more EVBs by lowering the selling price, and also to encourage Tto collect more used batteries. It can be argued that the manufacturer follows Vickers’ licensing principle, where the intention is non-profit maximization, rather than profit maximization.

### Impact of fairness and CSR coefficient on R decision making

**Figure 4:**
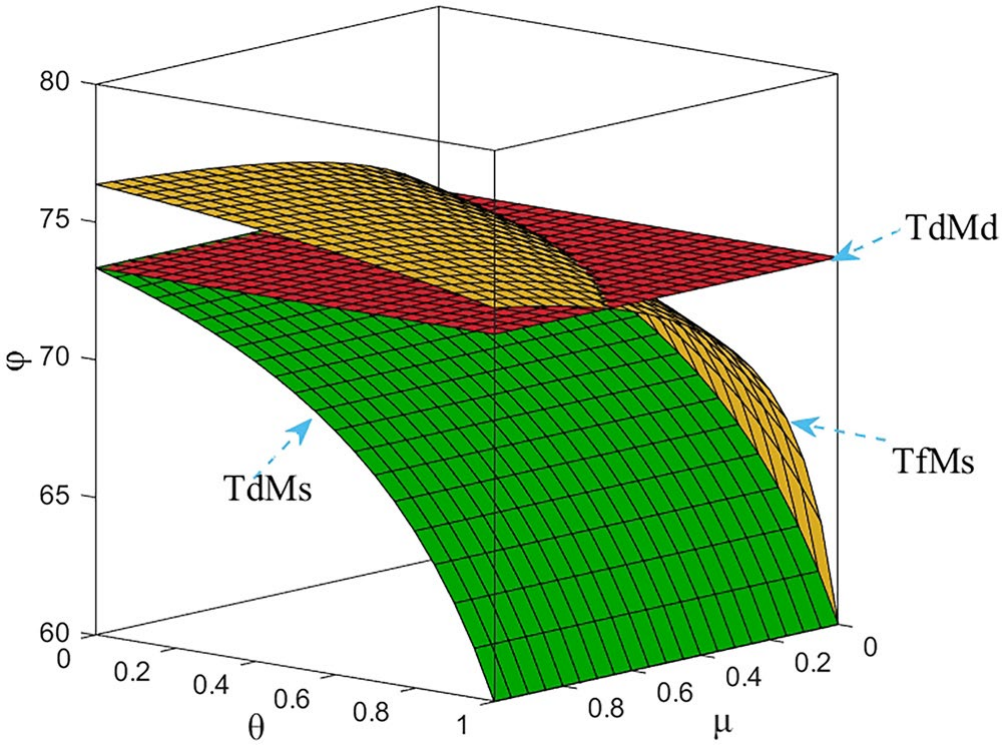
The impact of *μ* and *θ* on retail price.

**Figure 5:**
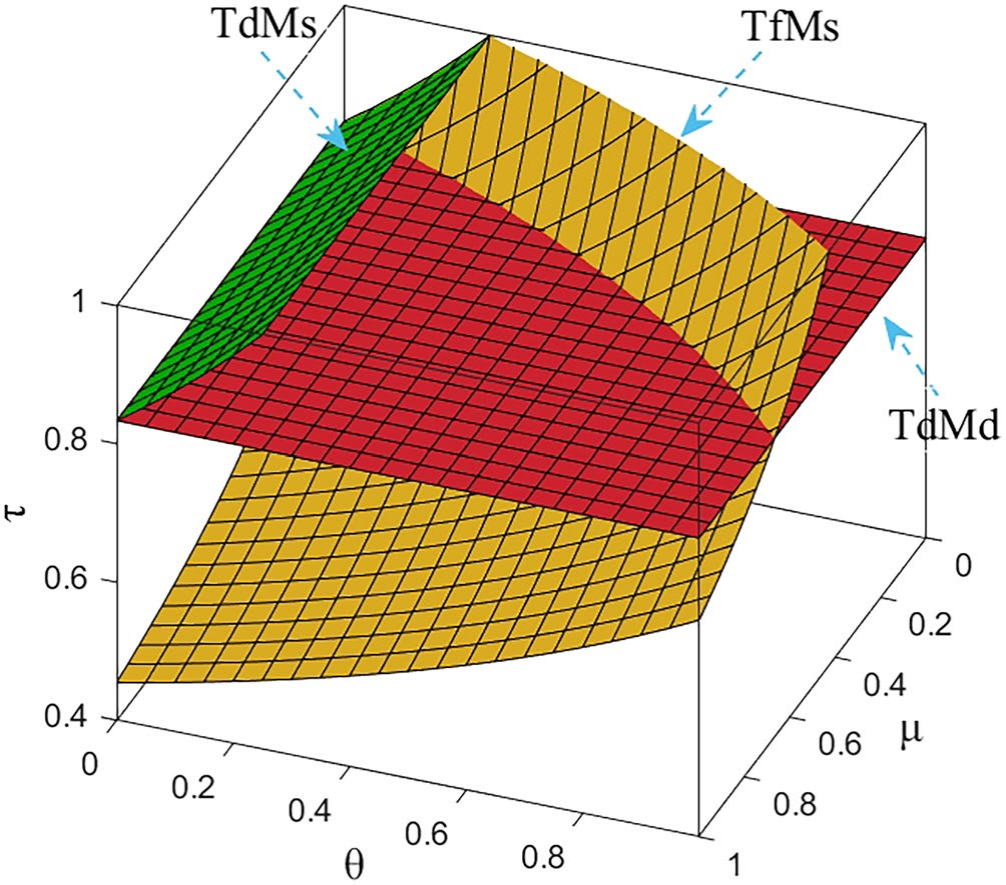
The impact of *μ* and *θ* on collecting rate.

**Figure 6:**
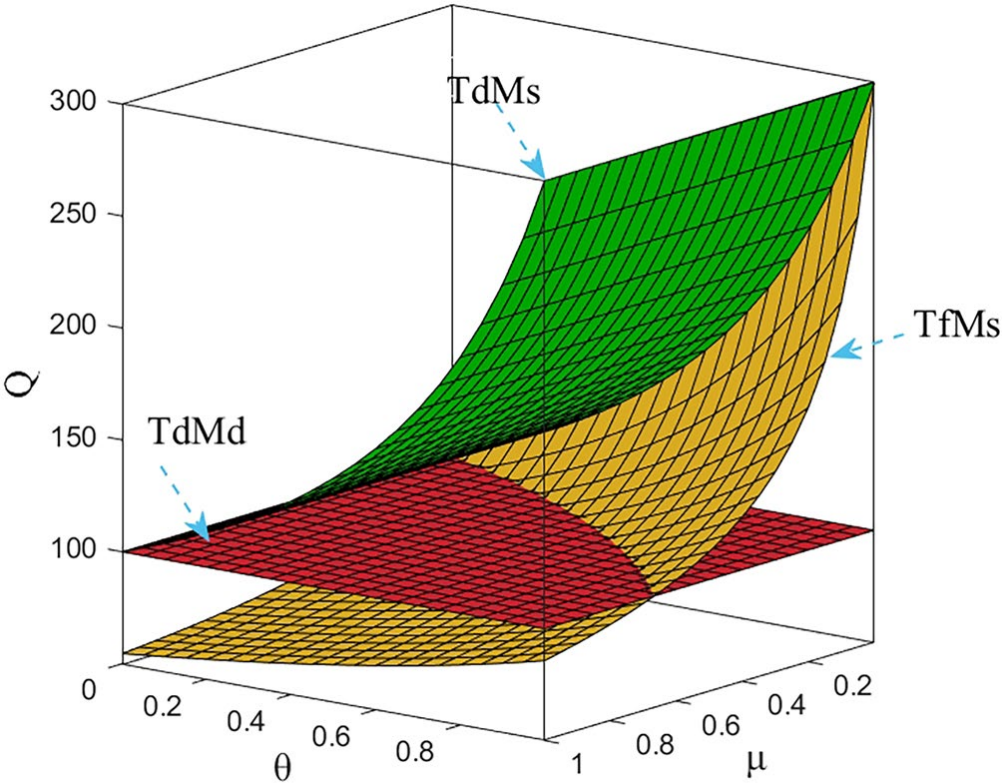
The impact of *μ* and *θ* on Q.

As shown in Fig. 4, when the fairness concern coefficient of T increases, the retail price also gradually increases under the influence of the change of M’s wholesale price, but the range of change is small in order to prevent the price from being too high and affecting the sales volume, but does not affect the share of R's profits in CLSC. In addition, Figs. 2, 3, 4, 5 and 6 further show that sales of power batteries can be effectively optimized with M considering CSR. As the CSR coefficient increases, the decrease in retail price further expands the market demand, and in addition, it can be seen from Fig. 6 that when *θ* =1, only under the influence of M's CSR, the market demand of EVBs is three times as large as the market demand in the model TdMd, and that when 0 ≤ *μ* ≤ 2/3, even if T generates a behavior of fairness concern, after M assumes CSR the market demand is also always greater than the market demand in TdMd. And in any case, R's profits always come first. Promoting NEVs instead of fuel vehicles by expanding the EVBs market is R's main responsibility.

### Impact of fairness and CSR coefficient on T decision making

It is clear from Fig. 5 that the collecting rate is proportional to the manufacturer’s CSR and has a specific range. When the manufacturer puts more weight on CSR, it incentivizes T to collect more used power batteries from customers by lowering its own wholesale price expands the demand for EVBs in the market, and benefiting both R and T at the same time, instead of increasing the purchase price of metal raw materials to benefit T alone. Therefore, the manufacturer's CSR affect the retail price decisions and the magnitude of market demand, as well as its decisions in the reverse supply chain. In addition, by numerical calculation, when *θ* = 1, ${\tau }_{d}^{S*}=2.5$, the collecting rate is three times as much as when there is no CSR, but obviously this is not realistic, so the collecting rate can only be taken to his maximum value of 1. On the other hand, when T does not satisfy the current profit distribution, the fluctuation of the collecting rate as well as the utility to himself is very large, and it can be seen from Fig. 10, that the utility of three enterprises shows a sharp decrease in the trend, but the utility gap between T and M gradually decreases, which shows that T's fairness concern plays a role, but in order to have a more balanced profit distribution, T does not hesitate to damage his own profit as well as that of the whole supply chain.

**Figure 7:**
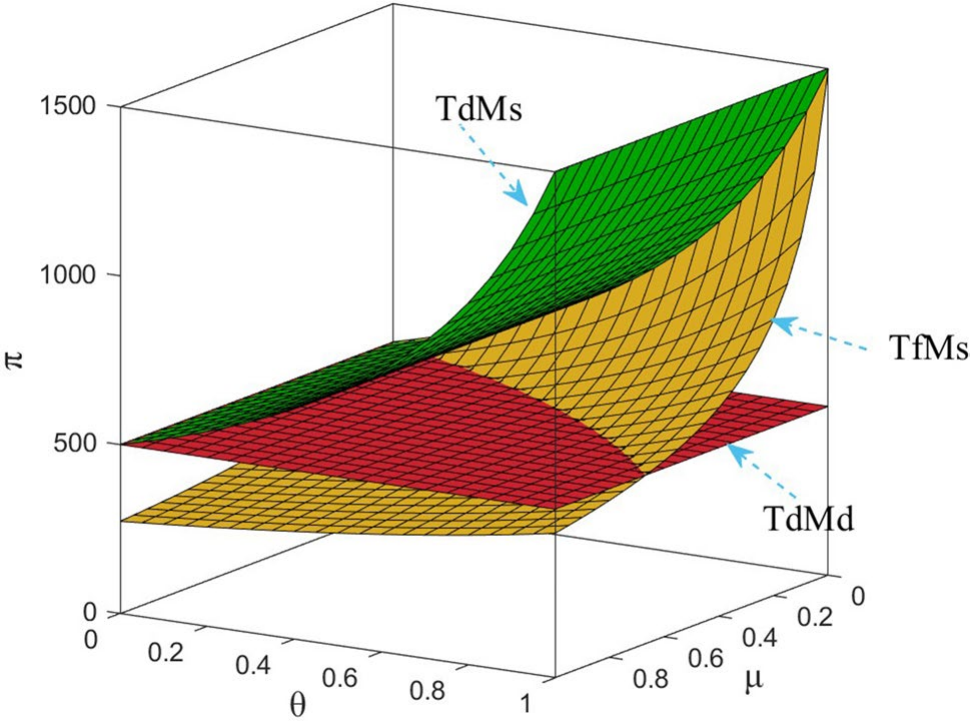
The impact of *μ* and *θ* on M’s profit.

**Figure 8:**
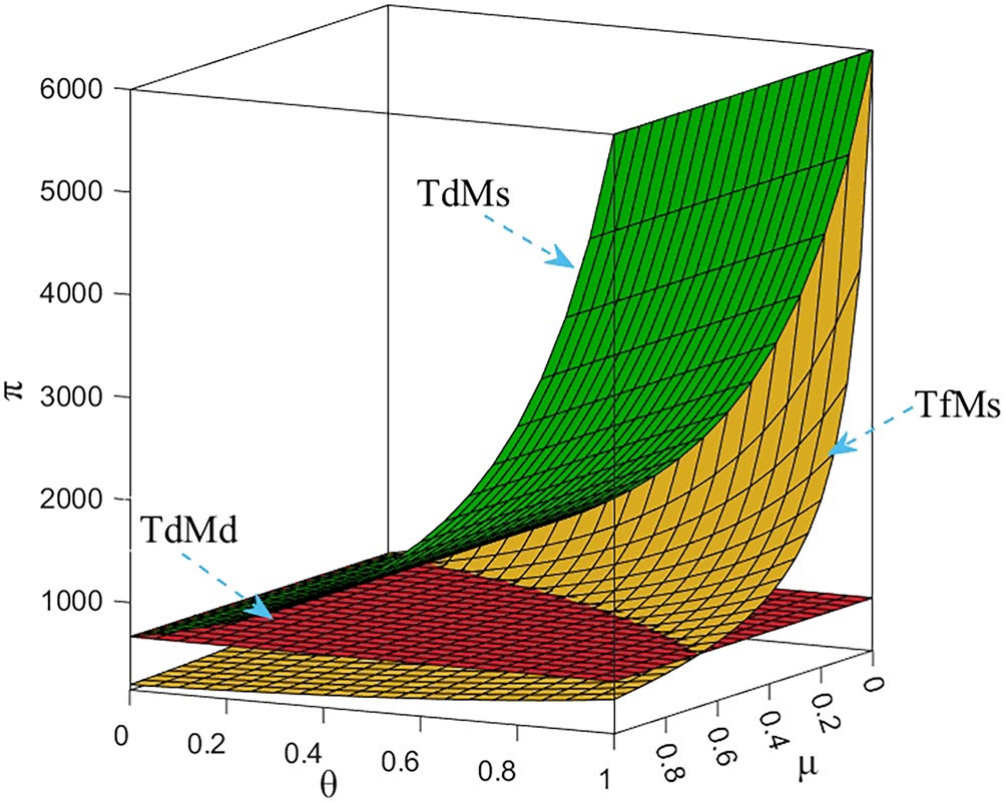
The impact of *μ* and θ on R’s profit.

**Figure 9:**
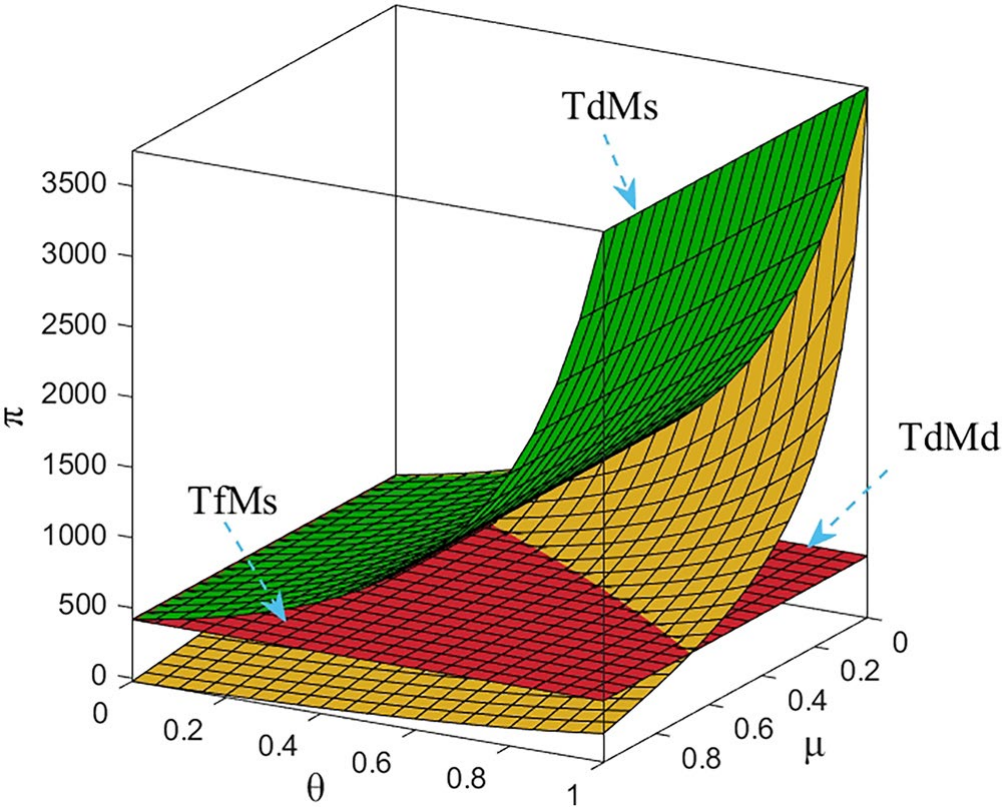
The impact of *μ* and θ on T’s profit.

**Figure 10:**
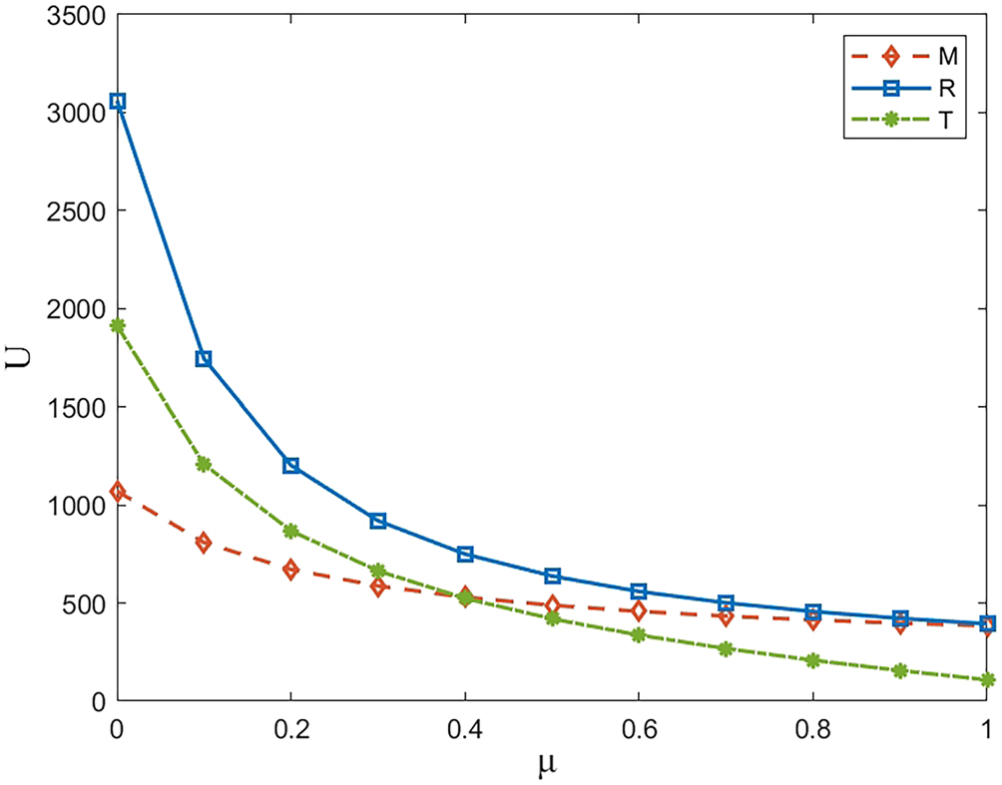
The impact of *μ* on profit in model TfMs.

The profit changes of the power battery closed-loop supply chain in the decentralized decision-making are shown in Figs. 7, 8, 9 and 10. It can be seen from Fig. 10: when T has fair concern behavior, with the increase of the degree of fair concern, the utility gap between T and other participants gradually decreases, and the decreasing trend of the effect is slower than that of M. However, at the same time, the utility of the three parties gradually decreases, which means that the profit of the whole supply chain is also decreasing, among which R is more sensitive to the fair concern coefficient and has the largest range of utility changes. It can be seen that T's fairness concern plays its due effect, preferring to penalize M and R by causing a loss of its own benefits in order to obtain what it considers to be a fair distribution of channel benefits. It can be further seen that taking CSR can effectively increase the profits of the green closed-loop supply chain and its members. When the fair concern coefficient and M's CSR coefficient satisfy certain conditions, the profits gained by M, R and T are higher than the profits under the decentralized decision-making with M aiming at profit maximization and T being fair and neutral. Therefore, for R, it is more desirable for M to assume CSR and make up for part of the loss.

### Analysis of the results of the cost-sharing compact

Based on the assumed values, the range of values for the fairness concern coefficient as well as the CSR coefficient can be calculated as:

$$0<{\phi }_{2}<\frac{2A(\theta -4)(1+\mu )-(1+\mu )(F-\Delta \lambda )J+\beta (F-\Delta \lambda {)}^{2}}{A(\theta -4)(1+\mu {)}^{2}}.$$

Within this range, we specify *μ* = 0.6, $\phi \in [\mathrm{0,0.12}]$. The results obtained after the harmonization of cost-sharing covenants are shown in Table 3.

**Table 3:** Summary of changes in contractual harmonization values.

* Φ*	0	0.02	0.04	0.06	0.08	0.1	0.12
${\omega }_{\phi \mu }^{S*}$	67.7863	67.5938	67.3884	67.1689	66.9338	66.6811	66.4091
${\sigma }_{\phi \mu }^{S*}$	34.3750	34.1209	33.8585	33.5872	33.3066	33.0163	32.7157
${\varphi }_{\phi \mu }^{S*}$	73.8931	73.7969	73.6942	73.5845	73.4669	73.3406	73.2045
${\tau }_{\phi \mu }^{S*}$	0.763	0.7880	0.8143	0.8424	0.8725	0.9048	0.9396
${Q}_{\phi \mu }^{S*}$	91.6031	93.0467	94.5867	96.2329	97.9968	99.8914	101.9320

**Figure 11:**
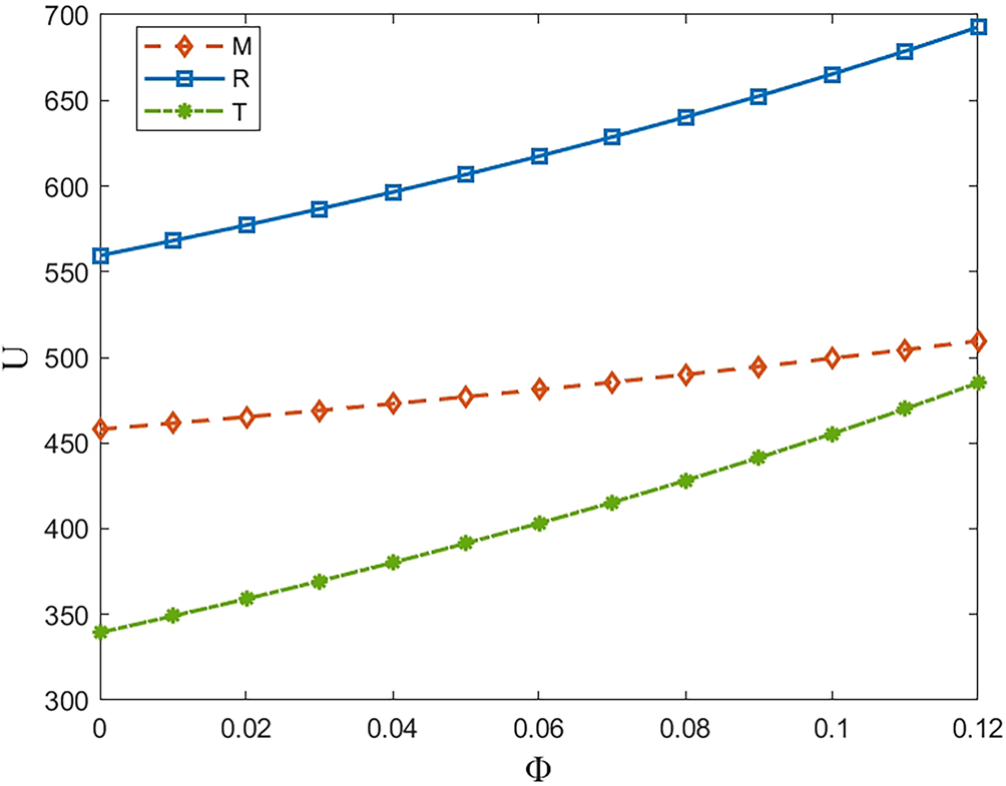
The impact of *ϕ* on profit in model TfMsc.

Through the simulation values in Table 3, we can visualize the effectiveness of the cost-sharing contract, the value of each decision variable with the increase of the coefficient shows a tendency to be more favorable to the enterprise and the consumer. According to the previous Theorem 3, only the concept of fairness concern will have an impact on the repurchase price. At the same time, we find that, the purchase price of metal raw materials did not increase accordingly. However, as can be seen from Fig. 11, although M bears part of the recovery cost for T, the profit gained by the three parties in the end on the basis of the decline in the wholesale price and the retail price is not reduced as a result of the decline in the wholesale price and the retail price, but rather, within a certain range as *φ* increases, and even for the downstream enterprises and even the entire supply chain to bring about a significant gain, the It can effectively reduce the sense of unfairness of T, thus promoting production and consumption of power batteries, and realizing the virtuous circle of economic and environmental development. Looking at the whole CLSC, when the profit of the whole enterprise increases sharply and the utility difference with M becomes smaller and smaller, the loss of T is not enough to mention.

## Summary and recommendations

### Conclusions

In this study, a closed-loop supply chain model is constructed to describe the manufacturing, recycling, dismantling, secondary use and remanufacturing processes of EVBs, taking into account the stepwise utilization of power batteries. Unlike other products, EVBs need to be dismantled from EVs when the capacity drops to 70–80%. However, batteries with a capacity of 20–80% can be used in projects such as energy storage as well as low-speed electric vehicles and other areas, while batteries with a capacity of less than 20% will be dismantled by specialized manufacturers to refine the chemical elements. Let more retired batteries utilize their residual value is the focus of current research. Unlike existing studies, most of the current studies on closed-loop supply chains for EVBs consider the impacts of recycling technologies^42^, government policies, and different recycling modes on enterprise decisions^43,44^, while ignoring the reality of profit distribution fairness in CLSC. It is not known that intra-firm competition has a great negative impact on the development of the closed-loop supply chain as a whole. On the one hand, this paper explores the impacts of CSR activities and the unfair behaviours of third-party recyclers in reality on the decision-making of each member of the closed-loop supply chain. Meanwhile, in order to solve the problem of benefit distribution among M, R and T, this paper designs a cost-sharing contract to better facilitate the recycling of EVBs. Finally, the results obtained in this paper and some of the key model parameters used are analyzed through numerical studies in order to validate the conclusions drawn in the previous section. The following results can be drawn from this paper:

1. M up to CSR can reduce the sense of unfairness of T and make a large number of retired batteries flow to the formal channel. When M undertakes CSR, R will reduce the sales price to encourage consumers to buy more power batteries, and at the same time the collection rate will gradually increase, and its value may exceed 1. However, this does not affect M's recycling price of metal materials, because stimulating the collection rate to fundamentally solve the profit mode of professional recycling is more effective than increasing the repurchase price, and it can stimulate the recycling motivation of T, which is conducive to reducing the environmental pollution of power batteries on the one hand, and increasing the profit of T on the other hand, and reducing its sense of unfairness. environment, on the other hand it can increase T's profit and reduce its sense of unfairness. In addition, the profits of all three parties are proportional to the CSR coefficient. Therefore, the socially responsible supply chain is more competitive than the traditional pure profit maximization channel, and the supply chain should tend to adopt the strategy of improving market competitiveness rather than insisting on the profit maximization strategy. This actually describes the power and necessity of using CSR in business practices.
2. T's fairness concerns are not conducive to the development of a closed-loop supply chain for power batteries. In fact, when the degree of fairness of the third party is relatively weak, the reduction of the profit of each member is larger, and the third party's profit share will increase, but at this time the wholesale price and retail price will increase, the third party has slack behavior, and the collection rate of power batteries decreases, which is unfavorable to the expansion of the market demand for EVBs and the recycling and reutilization of decommissioned batteries; when the degree of fairness is strong, but its behavior not only harms the other companies' interests, but also causes losses to itself and the interests of the whole supply chain. Become the enterprise with the lowest profit share. It can be seen that in order to ensure the fair distribution of channel profits, T would rather penalize each other than obtain a relatively fair result.
3. The cost-sharing pact resolves channel conflicts and get multiple wins. In fact, the coordination of CLSC is a strategy to consider how to respond to T's fairness concern in response to government policy. The cost-sharing ratio is directly proportional to T's fairness coefficient. The greater T's sense of unfairness, the more costs M will bear for it to reduce T's pressure, and at the same time, the higher the revenues of the three parties will be. By this way, it can effectively solve the internal contradiction of the channel and also make the business activities of the enterprise more flexible.

In the theoretical sense, this paper integrates M's CSR and T's fairness concern into the EVBs closed-loop supply chain system, which enriches the research on EVBs recycling management and provides a theoretical basis for the recycling and reuse of used power batteries. In the practical sense, considering the seriousness of the current waste power battery recycling problem, in order to effectively improve the collecting rate and reduce the harm to the environment and human beings. This paper proposes a new mode of solving the internal contradiction of cooperation operation of the channel. This model is an effective solution to the low recycling efficiency of existing waste power batteries and the low motivation of professional recycling and dismantling enterprises, and it is a reasonable and effective solution to the real recycling problem, which involves the ideas of collaboration and cost-sharing, and is also widely used in the recycling field.

### Strategies

Based on the above conclusions, we can get the following management insights: (1) The government should improve the policy system related to power battery recycling, consider the EVBs market situation and financial pressure, and subsidize it within the budget, so that the enterprises can take the initiative to undertake corporate social responsibility. Or it can directly formulate a policy to subsidize third-party recycling costs to reduce their recycling pressure and sense of unfairness. (2) EVBs manufacturer, as leader of CLSC, should not only play a leading role, but also take the initiative to shoulder social responsibility, because having CSR awareness can not only win a good social reputation and enhance the competitiveness of the company, but also promote the sustainable development of the EVBs closed-loop supply chain. Most importantly, it can make up for the loss caused by T’s fair concern behavior. In addition, it is necessary to strengthen cooperation with third-party collector, outsource dismantling and refining and other specialized businesses, and focus on the main business of EVBs manufacturing and remanufacturing. (3) R, as an "intermediary", is influenced by the cooperation between M and T, and can profit without paying much cost. Therefore, it is more important to harmonize the relationship between the M and T. It is also necessary to expand the sales business of EVBs, and at the same time to help T to collect more EVBs by means of trade-in and setting up recycling stations, so as to reduce the amount of them flowing into small workshops and to promote the maximization of resource utilization. (4) The third-party collector can finance from related enterprises and set up specialized R&D teams to reduce the pressure brought by too high cost of technical equipment. At the same time, they should look at the problem rationally, consider the loss brought by selfish behavior to themselves and the whole supply chain, and take the cooperative way to improve their own utility as far as possible. (5) Finally, the power battery closed-loop supply chain participants should strengthen communication and collaboration, closely observe each other's mentality and future decision-making trends, and respond flexibly to reduce internal contradictions and promote the benign development of the whole supply chain.

### Research limitations and future research trends

There are some limitations in this study. Firstly, this study is based on a single channel of recycling by a third-party collector, in fact, there are also multiple modes of recycling, such as M's own collecting, R's participation in collecting, and multi-channel collecting methods in the reverse supply chain of used power batteries. Secondly, this study only considers the third-party collector's concern about fairness. In fact, as a channel participant, the retailers' as well as other competitors' concern about fairness also exists. Lastly, this study only considered the cost-sharing contract in view of the high cost of third-party collecting, and did not consider whether there are other covenants that are more effective in coordinating this supply chain. 

### Supplementary Information

Supplementary Information.

## Data Availability

All data generated or analysed during this study are included in this published article and its supplementary information files.
