# Supplementary material for: Research on coordination of the NEV battery closed-loop supply chain considering CSR and fairness concerns in third-party recycling models
Source: Sci Rep. 2023 Dec 13;13:22172. doi: 10.1038/s41598-023-49047-8 (PMC10719397; doi:10.1038/s41598-023-49047-8)
Supplement: Supplementary file 1 — Supplementary Information. [file 41598_2023_49047_MOESM1_ESM.docx]

**Appendix**

**Appendix A.**

According to the order of the Stackelberg game, R and T are first analyzed, and by calculating the second-order derivative of $\pi_{dT}$ with respect to the recovery rate *τ*, and obtaining $\frac{\partial^{2}\pi_{dT}}{\partial\tau^{2}}=-A<0$, it can be seen that the profit function of T is a concave function with respect to the recovery rate, there is an optimal solution. Then the value of the collecting rate can be obtained by solving for the first-order derivative of $\pi_{dT}$ with respect to the collecting rate equal to 0:

$\tau_{d}=-\frac{(\alpha-\beta\varphi)(F-\lambda\sigma)}{A}$ (A.1)

Analyze the retailer and find the second-order partial derivative of $\pi_{dR}$ with respect to the retail price *φ*, to obtain $\frac{\partial\pi_{dR}}{\partial\varphi}=-2\beta<0$. Make its first-order derivative equal to 0 and solve the value of the retail price as:

$\varphi_{d}=-\frac{\alpha+\beta\omega}{2\beta}$ (A.2)

Substituting (A.1) and (A.2) into the profit function of M yields:

$\pi_{dM}=\frac{1}{2}(\alpha-\beta\varphi)(\omega-\frac{\lambda(\Delta-\sigma)(F-\lambda\sigma)(\alpha-\beta\omega)}{A}-c_{m})$ (A.3)

The Hessian matrix of $\pi_{dM}$ is

$$\left\{ \begin{aligned} &\frac{\partial^{2}\pi_{dM}}{\partial\sigma^{2}} \frac{\partial^{2}\pi_{dM}}{\partial\sigma\partial\omega} \\ &\frac{\partial^{2}\pi_{dM}}{\partial\omega\partial\sigma} \frac{\partial^{2}\pi_{dM}}{\partial\omega^{2}} \end{aligned} \right\}=\left[ \begin{aligned} &\text{ }-\frac{\lambda^{2}(\alpha-\beta\omega)^{2}}{2A}\text{ }-\frac{\beta\lambda(F+\lambda(\Delta-2\sigma))(\alpha-\beta\omega)}{2A} \\ &-\frac{\beta\lambda(F+\lambda(\Delta-2\sigma))(\alpha-\beta\omega)}{2A}\text{ }\frac{-2A\beta-\beta^{2}\lambda(\Delta-\sigma)(F-\lambda\sigma)}{2A} \end{aligned} \right]$$

The above matrix shows that $-\frac{\lambda^{2}(\alpha-\beta\omega)^{2}}{2A}<0$. The utility function of M is a joint concave function with respect to *ω* and *σ* only when $\left| H \right|>0$:

$$A\geq\frac{\beta((F+\lambda(\Delta-2\sigma))^{2}-\lambda(\Delta-2\sigma)(F-\lambda\sigma))}{2}$$

Finally, by finding the first order derivative of equation (A.3) with respect to and equal to 0, the system of simultaneous equations, the optimal solution for the manufacturer's wholesale *ω* and purchase prices *σ* can be obtained as:

$\omega_{d}^{*}=\frac{\alpha(-4A+\beta(F-\Delta\lambda)^{2})-4A\beta c_{m}}{\beta(-8A+\beta(F-\Delta\lambda)^{2})}$ (A.4)

$\sigma_{d}^{*}=\frac{F+\Delta\lambda}{2\lambda}$ (A.5)

Substituting the optimal solution equations (A.4) and (A.5) into (A.1), (A.2), and (A.3) yields optimal solutions for the collecting rate, market demand and retail price:

$\tau_{d}^{*}=\frac{(F-\Delta\lambda)J}{-8A+\beta(F-\Delta\lambda)^{2}}$ (A.6)

$\varphi_{d}^{*}=\frac{\alpha}{2\beta}+\frac{\alpha(-4A+\beta(F-\Delta\lambda)^{2})-4A\beta c_{m}}{\beta(-16A+2\beta(F-\Delta\lambda)^{2})}$ (A.7)

$Q_{d}^{*}=\frac{2AJ}{8A-\beta(F-\Delta\lambda)^{2}}$ (A.8)

Finally, the optimal solutions of all decision variables are substituted into Eqs. (1), (2), and (3) to obtain the utility function of each member in the closed-loop supply chain:

$$\pi_{dM}^{*}=-\frac{AJ^{2}}{\beta(-8A+\beta(F-\Delta\lambda)^{2})}$$

$$\pi_{dR}^{*}=\frac{4A^{2}J^{2}}{\beta(-8A+\beta(F-\Delta\lambda)^{2})^{2}}$$

$$\pi_{dT}^{*}=\frac{A(F-\Delta\lambda)^{2}J^{2}}{2(-8A+\beta(F-\Delta\lambda)^{2})^{2}}$$

**Appendix B.**

From Proposition 1 we have: $\pi_{dM}^{*}-\pi_{dR}^{*}=-\frac{A(4A-\beta(F-\Delta\lambda)^{2})J^{2}}{\beta(-8A+\beta(F-\Delta\lambda)^{2})^{2}}$, because of *J* > 0，and we can prove that $\beta(F-\Delta\lambda)^{2}>4A$, thus $\pi_{dM}^{*}-\pi_{dR}^{*}<0$*.* Similarly, we have $\pi_{dR}^{*}-\pi_{dT}^{*}=-\frac{AJ^{2}}{2\beta(-8A+\beta(F-\Delta\lambda)^{2})}>0$, $\pi_{dM}^{*}-\pi_{dT}^{*}=\frac{A(16A-3\beta(F-\Delta\lambda)^{2})J^{2}}{2\beta(-8A+\beta(F-\Delta\lambda)^{2})^{2}}>0$.

Because of $\Delta\pi_{d}=\pi_{dM}^{*}-\pi_{dT}^{*}=A\tau+(\alpha-\beta\varphi)(\lambda-(\Delta-\sigma)+(F-\lambda\sigma))$，Substituting (A.5), (A.6) and (A.7) into the above equation, it can be known that $\frac{\partial\Delta\pi}{\partial\tau}=A\tau>0$.

**Appendix C.**

The solution process is similar to Appendix A，because of $\frac{\partial^{2}V_{T}^{S}}{\partial\tau^{2}}=-A<0$，$\frac{\partial^{2}V_{R}^{S}}{\partial\varphi^{2}}=-2\beta<0$, There is a unique optimal solution for retail price and recovery rate. The optimal solution is then obtained by a first-order derivative equal to zero:

$\tau_{d}^{S}=-\frac{(F-\lambda\sigma)(\alpha-\beta\varphi)}{A}$ (C.1)

$\varphi_{d}^{S}=\frac{\alpha+\beta\omega}{2\beta}$ (C.2)

Substituting (C.1) and (C.2) into Eq. (4), the Hessian matrix is obtained as:

$$\left| H \right|=\left\{ \begin{aligned} &\frac{\partial^{2}V_{M}}{\partial\sigma^{2}} \frac{\partial^{2}V_{M}}{\partial\sigma\partial\omega} \\ &\frac{\partial^{2}V_{M}}{\partial\omega\partial\sigma} \frac{\partial^{2}V_{M}}{\partial\omega^{2}} \end{aligned} \right\}=\left[ \begin{aligned} &\text{ }-\frac{2\lambda^{2}(\alpha+\frac{1}{2}(\alpha-\beta\omega))^{2}}{A}\text{ }-\frac{\beta\lambda(F+\lambda(\Delta-2\sigma))(\alpha-\beta\omega)}{2A} \\ &-\frac{\beta\lambda(F+\lambda(\Delta-2\sigma))(\alpha-\beta\omega)}{2A}\text{ }\frac{\beta(A(\theta-4)-2\beta\lambda(\Delta-\sigma)(F-\lambda\sigma))}{4A} \end{aligned} \right]$$

Clearly， $\left| H \right|_{11}=-\frac{2\lambda^{2}(\alpha+\frac{1}{2}(\alpha-\beta\omega))^{2}}{A}<0$ . For the manufacturer's utility function to be a joint concave function with respect to *ω* and *σ*, it is necessary that $\left| H \right|>0$, thus $A\geq-\frac{\beta(F-\Delta\lambda)^{2}}{2(\theta-4)}$
。Finally, according to $\frac{\partial V_{M}^{S}}{\partial\omega}=0$,$\frac{\partial V_{M}^{S}}{\partial\sigma}=0$，taking the optimal solution to the wholesale and repurchase prices：

$$\omega_{d}^{S*}=\frac{2A\alpha(\theta-2)+\alpha\beta(F-\Delta\lambda)^{2}-4A\beta c_{m}}{\beta(2A(\theta-4)+\beta(F-\Delta\lambda)^{2})}$$

$$\sigma_{d}^{S*}=\frac{F+\Delta\lambda}{2\lambda}$$

Therefore, the collecting rate, retail price and market demand are obtained:

$$\varphi_{d}^{S*}=\frac{\alpha}{2\beta}+\frac{2A\alpha(\theta-2)+\alpha\beta(F-\Delta\lambda)^{2}-4A\beta c_{m}}{4A\beta(\theta-4)+2\beta^{2}(F-\Delta\lambda)^{2}}$$

$$\tau_{d}^{S*}=\frac{(F-\Delta\lambda)J}{2A(\theta-4)+\beta(F-\Delta\lambda)^{2}}$$

$$Q_{d}^{S*}=-\frac{2AJ}{2A(\theta-4)+\beta(F-\Delta\lambda)^{2}}$$

Finally, substituting the resulting equilibrium solutions into equations (5), (6) and (7), the profits of the three parties are respectively：

$$V_{M}^{S*}=-\frac{AJ^{2}}{\beta(2A(\theta-4)+\beta(F-\Delta\lambda)^{2})}$$

$$V_{R}^{S*}=\frac{4A^{2}J^{2}}{\beta(2A(\theta-4)+\beta(F-\Delta\lambda)^{2})^{2}}$$

$$V_{T}^{S*}=\frac{A(F-\Delta\lambda)^{2}J^{2}}{2(2A(\theta-4)+\beta(F-\Delta\lambda)^{2})^{2}}$$

**Appendix D.**

The relationship between each decision variable and the profit and CSR coefficients of the three parties is as follows:

$\frac{\partial V_{M}^{S*}}{\partial\theta}=\frac{2A^{2}J^{2}}{\beta(2A(\theta-4)+\beta(F-\Delta\lambda))^{2}}>0$. Because of $\theta-4<0$, $\left| 2A(\theta-4) \right|>\beta(F-\Delta\lambda)^{2}$, there is $\frac{\partial V_{R}^{S*}}{\partial\theta}=-\frac{16A^{3}J^{2}}{\beta(2A(\theta-4)+\beta(F-\Delta\lambda)^{2})^{3}}>0$. $\frac{\partial V_{T}^{S*}}{\partial\theta}=\frac{2A^{2}(F-\Delta\lambda)^{2}J^{2}}{(2A(\theta-4)+\beta(F-\Delta\lambda)^{2})^{3}}>0$.

$\frac{\partial\omega_{d}^{S*}}{\partial\theta}=-\frac{8\beta A^{2}J}{(2A(\theta-4)+\beta(F-\Delta\lambda))^{2}}<0$. $\frac{\partial\sigma_{d}^{S*}}{\partial\theta}=0$. $\frac{\partial\varphi_{d}^{S*}}{\partial\theta}=-\frac{4A^{2}\beta J}{(2A(\theta-4)+\beta(F-\Delta\lambda)^{2})^{2}}<0$.

$\frac{\partial\tau_{d}^{S*}}{\partial\theta}=-\frac{2A(F-\Delta\lambda)J}{(2A(\theta-4)+\beta(F-\Delta\lambda)^{2})^{2}}<0$. $\frac{\partial Q_{d}^{S*}}{\partial\theta}=\frac{4A^{2}J}{(2A(\theta-4)+\beta(F-\Delta\lambda)^{2})^{2}}>0$

**Appendix E.**

Similarly，first take the second order derivatives of the T and R decision variables，there is$\frac{\partial^{2}U_{T}}{\partial\tau^{2}}=-A<0$, $\frac{\partial^{2}U_{R}}{\partial\varphi^{2}}=-2\beta<0$, Then solve for the recovery rate as well as the retail price by a first order derivative equal to zero：

$\tau_{\mu}^{S}=-\frac{(F+\Delta\lambda\mu-\lambda(1+\mu)\sigma)(\alpha-\beta\varphi)}{A}$ （E.1）

$\varphi_{\mu}^{S}=\frac{\alpha+\beta\omega}{2\beta}$ （E.2）

Substituting (E.2) and (E.2) into equation (5) solves for the mixed second-order derivative of the manufacturer's relevant wholesale and repurchase prices:

$$\left| H \right|=\left\{ \begin{aligned} &\frac{\partial^{2}U_{M}}{\partial\sigma^{2}} \frac{\partial^{2}U_{M}}{\partial\sigma\partial\omega} \\ &\frac{\partial^{2}U_{M}}{\partial\omega\partial\sigma} \frac{\partial^{2}U_{M}}{\partial\omega^{2}} \end{aligned} \right\}=\left[ \begin{aligned} &\text{ }-\frac{\lambda^{2}(1+\mu)(\alpha-\beta\omega)^{2}}{2A}\text{ }\frac{\lambda(F+\lambda(\Delta+2\Delta\mu-2(1+\mu)\sigma))(\alpha-\beta\omega)^{2}}{4A} \\ &\frac{\lambda(F+\lambda(\Delta+2\Delta\mu-2(1+\mu)\sigma))(\alpha-\beta\omega)^{2}}{4A}\text{ }-\beta+\frac{\theta}{4}-\frac{\beta^{2}\lambda(\Delta-\sigma)(F+\lambda\Delta\mu-\lambda(1+\mu)\sigma)}{2A} \end{aligned} \right]$$

Because of $\left| H \right|_{11}= -\frac{\lambda^{2}(1+\mu)(\alpha-\beta\omega)^{2}}{2A}<0$. For the manufacturer's utility function to be a joint concave function with respect to *ω* and *σ*, it is necessary that $\left| H \right|>0$，$A\geq-\frac{\beta(F-\Delta\lambda)^{2}}{2(\theta-4)(1+\mu)}$. Finally, according to $\frac{\partial U_{M}^{S}}{\partial\omega}=0$, $\frac{\partial U_{M}^{S}}{\partial\sigma}=0$，solve the optimal solution for the wholesale price and the repurchase price:

$$\omega_{\mu}^{S*}=\frac{2A\alpha(\theta-2)(1+\mu)+\alpha\beta(F-\Delta\lambda)^{2}-4A\beta c_{m}(1+\mu)}{\beta(2A(\theta-4)(1+\mu)+\beta(F-\Delta\lambda)^{2})}$$

$$\sigma_{\mu}^{S*}=\frac{F+\Delta\lambda(1+2\mu)}{2\lambda(1+\mu)}$$

Thus the recovery rate, retail price, marketing effort and market demand are obtained:

$$\varphi_{\mu}^{S*}=\frac{\alpha}{2\beta}+\frac{2A\alpha(\theta-2)(1+\mu)+\alpha\beta(F-\Delta\lambda)^{2}-4A\beta(1+\mu)c_{m}}{4A\beta(\theta-4)(1+\mu)+2\beta^{2}(F-\Delta\lambda)^{2}}$$

$$\tau_{\mu}^{S*}=\frac{(F-\Delta\lambda)(1+\mu)J}{2A(\theta-4)(1+\mu)+\beta(F-\Delta\lambda)^{2}}$$

$$Q_{\mu}^{S*}=-\frac{2AJ(1+\mu)}{2A(\theta-4)(1+\mu)+\beta(F-\Delta\lambda)^{2}}$$

Substituting the resulting equilibrium solutions into equations (2), (4) and (5), the final utility of the three parties is:

$$U_{M}^{S*}=-\frac{A(1+\mu)J^{2}}{\beta(\beta(F-\Delta\lambda)^{2}+2A(\theta-4)(1+\mu))}$$

$$U_{R}^{S*}=\frac{4(1+\mu)^{2}A^{2}J^{2}}{\beta(2A(\theta-4)(1+\mu)+\beta(F-\Delta\lambda)^{2})^{2}}$$

$$U_{T}^{S*}=\frac{A(1+\mu)J^{2}((1+5\mu)\beta(F-\Delta\lambda)^{2}+4A(\theta-4)\mu(1+\mu))}{2\beta(2A(\theta-4)(1+\mu)+\beta(F-\Delta\lambda)^{2})^{2}}$$

**Appendix F.**

Find the first-order derivatives with respect to θ and *μ* for the decision variables and utility, respectively.：

$\frac{\partial\omega_{\mu}^{S*}}{\partial\mu}=\frac{4A(F-\Delta\lambda)^{2}J}{(\beta(F-\Delta\lambda)^{2}+2A(\theta-4)(1+\mu))^{2}}>0$. $\frac{\partial\sigma_{\mu}^{S*}}{\partial\mu}=-\frac{F-\Delta\lambda}{2\lambda(1+\mu)^{2}}>0$.

$\frac{\partial\varphi_{\mu}^{S*}}{\partial\mu}=\frac{2A(F-\Delta\lambda)^{2}J}{(\beta(F-\Delta\lambda)^{2}+2A(\theta-4)(1+\mu))^{2}}>0$. $\frac{\partial\tau_{\mu}^{S*}}{\partial\mu}=\frac{\beta(F-\Delta\lambda)^{3}J}{(\beta(F-\Delta\lambda)^{2}+2A(\theta-4)(1+\mu))^{2}}<0$.

$\frac{\partial Q_{\mu}^{S*}}{\partial\mu}=-\frac{2A\beta(F-\Delta\lambda)^{2}J}{(\beta(F-\Delta\lambda)^{2}+2A(\theta-4)(1+\mu))^{2}}<0$. $\frac{\partial U_{M}^{S*}}{\partial\mu}=-\frac{A(F-\Delta\lambda)^{2}J^{2}}{(\beta(F-\Delta\lambda)^{2}+2A(\theta-4)(1+\mu))^{2}}<0$.

$\frac{\partial U_{R}^{S*}}{\partial\mu}=-\frac{8(1+\mu)A^{2}(F-\Delta\lambda)^{2}J^{2}}{(\beta(F-\Delta\lambda)^{2}+2A(\theta-4)(1+\mu))^{3}}<0$.

$\frac{\partial U_{T}^{S*}}{\partial\mu}=-\frac{A(12A\beta(\theta-4)(1+\mu)^{2}(F-\Delta\lambda)^{2}+8A^{2}(\theta-4)^{2}(1+\mu)^{3}+2\beta^{2}(F-\Delta\lambda)^{4}(3+5\mu))J^{2}}{2\beta(\beta(F-\Delta\lambda)^{2}+2A(\theta-4)(1+\mu))^{3}}<0$.

$\frac{\partial\omega_{\mu}^{S*}}{\partial\theta}=-\frac{8A^{2}(1+\mu)^{2}J}{\beta(\beta(F-\Delta\lambda)^{2}+2A(\theta-4)(1+\mu))^{2}}<0$. $\frac{\partial\sigma_{\mu}^{S*}}{\partial\theta}=0$. $\frac{\partial\tau_{\mu}^{S*}}{\partial\theta}=-\frac{2A(F-\Delta\lambda)(1+\mu)^{2}J}{(\beta(F-\Delta\lambda)^{2}+2A(\theta-4)(1+\mu))^{2}}>0$. $\frac{\partial\varphi_{\mu}^{S*}}{\partial\theta}=-\frac{4A^{2}(1+\mu)^{2}J}{\beta(\beta(F-\Delta\lambda)^{2}+2A(\theta-4)(1+\mu))^{2}}<0$. $\frac{\partial Q_{\mu}^{S*}}{\partial\theta}=-\frac{4A^{2}(1+\mu)^{2}J}{(\beta(F-\Delta\lambda)^{2}+2A(\theta-4)(1+\mu))^{2}}>0$.

$\frac{\partial U_{M}^{S*}}{\partial\theta}=\frac{2A^{2}(1+\mu)^{2}J^{2}}{\beta(\beta(F-\Delta\lambda)^{2}+2A(\theta-4)(1+\mu))^{2}}>0$. $\frac{\partial U_{R}^{S*}}{\partial\theta}=-\frac{16(1+\mu)^{3}J^{2}}{\beta(\beta(F-\Delta\lambda)^{2}+2A(\theta-4)(1+\mu))^{3}}>0$.

$\frac{\partial U_{T}^{S*}}{\partial\theta}=\frac{2A^{2}(1+\mu)^{2}(-\beta(F-\Delta\lambda)^{2}+8A\mu-2\mu(A\theta+2\beta(F-\Delta\lambda)^{2})-2A(\theta-4)\mu^{2})J^{2}}{\beta(\beta(F-\Delta\lambda)^{2}+2A(\theta-4)(1+\mu))^{3}}>0$

**Appendix G.**

The solution process is similar to Appendices A, C, and E. Note:

$C_{1}=2(\phi(1+\mu)^{2}-2\mu-1)+(1+\mu)(\phi\mu+\phi-1)$；$C_{2}=A(\theta-2)(1+\mu)(\phi\mu+\phi-1)^{2}$；$C_{2}=(F-\Delta\lambda)^{2}(\phi\mu+\phi-1)$

**Appendix H.**

When $\theta=1$，$Q_{\mu}^{S*}=-\frac{150（1+\mu）}{-0.5-3\mu}$，$Q_{d}^{*}=100$，let $Q_{\mu}^{S*}=-\frac{150（1+\mu）}{-0.5-3\mu}>100$, solve for $\mu=\frac{2}{3}$.
